# Review and Meta‐Analysis: SARS‐CoV‐2 and Enveloped Virus Detection in Feces and Wastewater

**DOI:** 10.1002/cben.202100039

**Published:** 2022-03-03

**Authors:** Charlotte Twigg, Jannis Wenk

**Affiliations:** ^1^ University of Bath Department of Chemical Engineering and Water Innovation and Research Centre (WIRC@Bath) Claverton Down BA2 7AY Bath Somerset United Kingdom

**Keywords:** COVID‐19, Real‐time reverse transcription polymerase chain reaction, SARS‐CoV‐2, Virus transmission, Wastewater‐based epidemiology

## Abstract

Detection and quantification of viruses supplies key information on their spread and allows risk assessment for public health. In wastewater, existing detection methods have been focusing on non‐enveloped enteric viruses due to enveloped virus transmission, such as coronaviruses, by the fecal‐oral route being less likely. Since the beginning of the SARS‐CoV‐2 pandemic, interest and importance of enveloped virus detection in wastewater has increased. Here, quantitative studies on SARS‐CoV‐2 occurrence in feces and raw wastewater and other enveloped viruses via quantitative real‐time reverse transcription polymerase chain reaction (RT‐qPCR) during the early stage of the pandemic until April 2021 are reviewed, including statistical evaluation of the positive detection rate and efficiency throughout the detection process involving concentration, extraction, and amplification stages. Optimized and aligned sampling protocols and concentration methods for enveloped viruses, along with SARS‐CoV‐2 surrogates, in wastewater environments may improve low and variable recovery rates providing increased detection efficiency and comparable data on viral load measured across different studies.

## Graphical abstract

This review provides an overview on most recent quantitative studies on SARS‐CoV‐2 occurrence in feces and raw wastewater including a statistical meta‐analysis on enveloped virus detection by quantitative real‐time reverse transcription polymerase chain reaction (RT‐qPCR), virus concentration, extraction, and amplification efficiency.

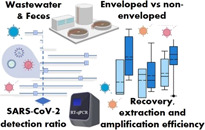

## 1 Introduction

The recent outbreak of the severe acute respiratory syndrome coronavirus 2 (SARS‐CoV‐2) pandemic has led to almost 400 million cases and more than 5.7 million deaths worldwide as of February 08, 2022 1. Unprecedented global consequences to public health systems, economies, and societies have highlighted the need for research on the fate of viruses in ecosystems and anthropogenic environments. Monitoring viral contamination in a community remains an important focus of understanding the extent of virus behaviour and transmission 2. Enveloped viruses, such as coronaviruses, are assumed to pose a low threat in wastewater due to their fragility.

Monitoring viruses involves complex, costly, and time‐consuming detection methods 3. A review on the detection efficacy for enveloped viruses in wastewater is necessary for insight into the accuracy of their detection and to allow a comparative overview across the various detection protocols applied. While enveloped viruses are structurally less stable compared to non‐enveloped viruses, employing the same detection methods for both types in the wastewater matrix may provide inaccurate reflection of virus concentrations.

This review focuses on summarizing the findings for the presence of SARS‐CoV‐2 in feces and raw wastewater in the early stages of the pandemic, in addition to reviewing efficiency of virus detection by quantitative real‐time reverse transcription polymerase chain reaction (RT‐qPCR) including virus concentration, extraction, and amplification steps from raw wastewater. Efficiency of each stage in the detection process is reviewed, with a specific focus on SARS‐CoV‐2 and surrogates.

The review is arranged into five sections. Section Sect. 2 provides a general introduction on viruses, their fate in wastewater and virus detection methods with a focus on SARS‐CoV‐2 and RT‐qPCR. Section Sect. 3 describes data search and evaluation strategies, including statistical analysis. Section Sect. 4 presents the results of the meta‐analyses for the detection of SARS‐CoV‐2 in feces and raw wastewater. Section Sect. 5 presents an analysis for different process control viruses by comparison of recovery, extraction, and amplification efficiency prior to RT‐qPCR evaluation, followed by general conclusions.

## 2 Virus Taxonomy, Detection, and Fate in Wastewater

**Figure 1:**
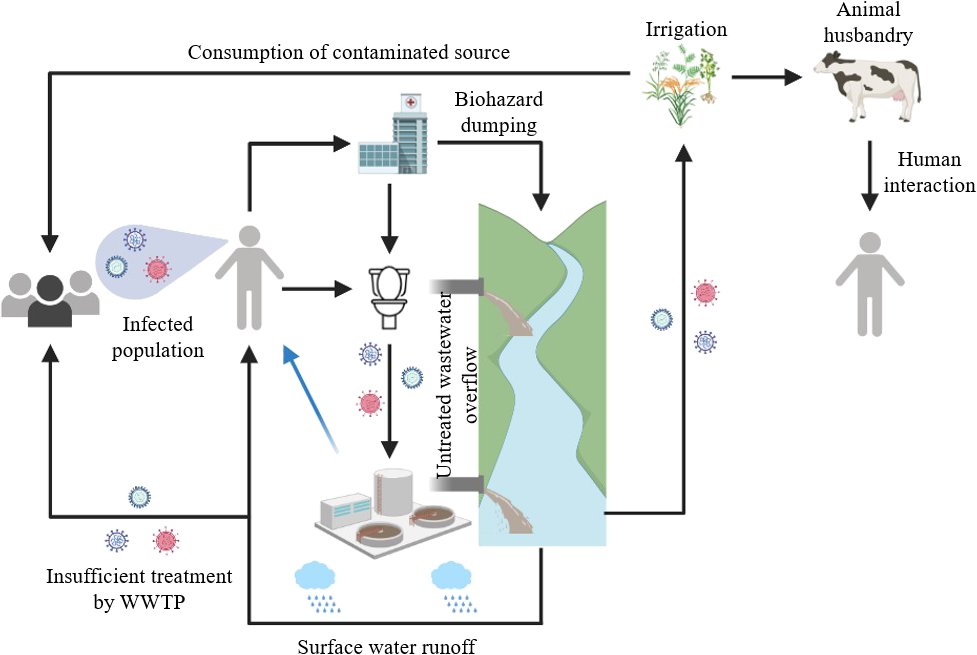
Schematic representation of pathways for virus transmission through water environments. Created from Biorender.com 13. The blue arrow signifies aerosol transmission. Adapted from 14.

Viruses are microscopic, obligate intracellular parasites that exhibit great diversity in shape and size, ranging between 10–400 nm 4, as well as genome structure, chemical composition, reproduction, and range of host species 5. Viruses consist of proteins, carbohydrates, and lipids. They have a basic nucleocapsid structure composed of nucleic acid enclosed within a virus‐coded protein capsid, responsible for controlling the host cell recognition and binding mechanism 6, 7, 8. Human pathogenic viruses may enter the water cycle via various point sources such as sewer systems and wastewater treatment plants (WWTPs), and non‐point sources (Fig. 1) 9. Previous research on virological removal and inactivation of viruses in WWTPs has focused primarily on non‐enveloped enteric viruses that are highly abundant in wastewater and readily transmitted via the fecal‐oral route 10. While enveloped viruses are fragile and transmitted through bodily fluids 11, their detection in wastewater may provide important epidemiological information 12.

Detection of viruses, including SARS‐CoV‐2 in sewage and wastewater via RT‐qPCR:

Coronaviruses are airborne, and primarily transmitted via respiratory droplets 15, 16. However, viral RNA can remain stable in stool samples 17 and infectious routes via aerosolization of fecal waste particles shown for coronaviruses (SARS‐CoV‐1) 18, 19, 20 and surrogates is plausible 21. Conventional wastewater treatment inactivates and removes SARS‐CoV‐2 22, 23, 24, 25, 26, 27, 28, 29. Virus reduction from untreated wastewater to tertiary treated effluents typically ranges between 2–3 log_10_ 30. The load of SARS‐CoV‐2 in feces of infected patients ranges between 10^4^–10^8^ copies L^−1^, and the concentration is reduced to 10^2^–10^6.5^ copies L^−1^ after mixing in sewage 31.

While infection risk of SARS‐CoV‐2 in wastewater is negligibly low, virus monitoring in sewage and wastewater is useful for wastewater‐based epidemiology (WBE) applications, complementary to clinical surveillance 32. WBE utilizes wastewater sampling to monitor the real‐time health status of a population within a sewage catchment area 33, and as early‐warning, detection methods to predict viral outbreaks 23, 34.

**Figure 2:**
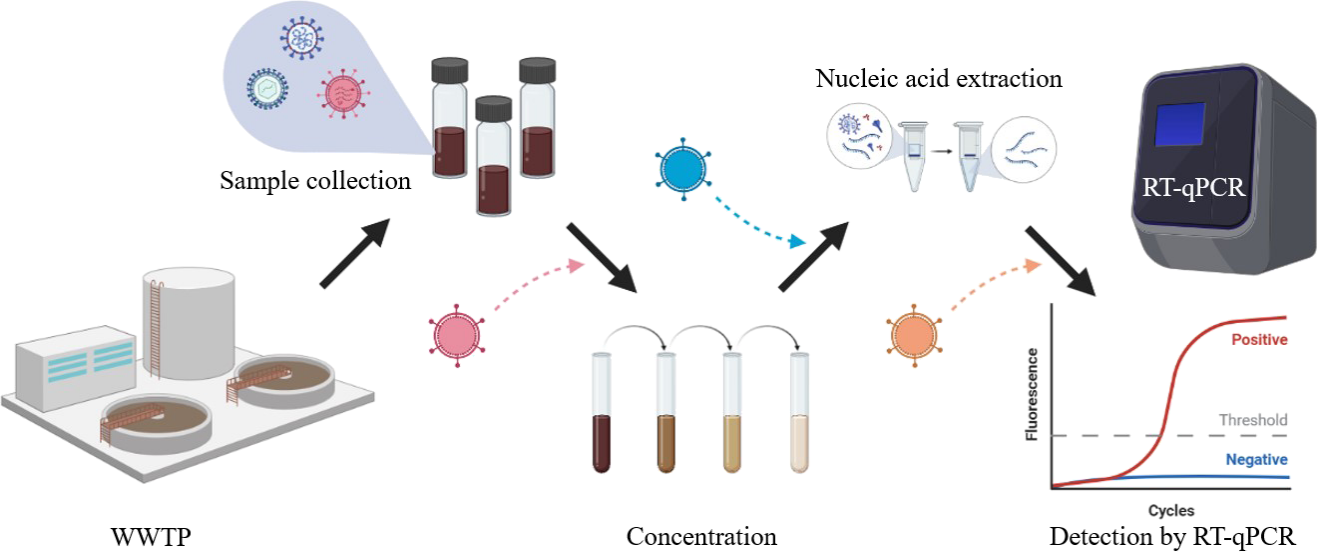
Wastewater sampling preparation and detection workflow for pathogens via RT‐qPCR. Whole process control (WPC), molecular process control (MPC), and RT‐qPCR control denote the quality control viruses that are inoculated prior to the detection step to measure recovery, extraction, and amplification efficiency, respectively. Adapted from 38.

Fig. 2 shows the RT‐qPCR sampling preparation and detection workflow for pathogens, including viruses in wastewater. The viral nucleic acid is concentrated, extracted, and amplified via RT‐qPCR to determine the viral concentration. Techniques to concentrate wastewater samples are required due to significantly lower target analyte concentration compared to urine or feces. For SARS‐CoV‐2 detection in wastewater, the most frequently used concentration methods include ultrafiltration, electronegative membrane filtration, polyethylene glycol (PEG) precipitation, flocculation, and ultracentrifugation 35, and were initially designed for enteric, non‐enveloped species 36. To determine the recovery efficiency of the concentration step, whole process controls (WPCs) are used to estimate the ratio between the concentration of virus detected and the concentration of a control virus spiked into the sample. Extraction efficiency is estimated with molecular process controls (MPCs), and RT‐qPCR controls are used to evaluate inhibitors that decrease amplification efficiency 37. These controls are typically surrogate viruses that represent the target virus detected in the process steps defined in Fig. 2.

**Figure 3:**
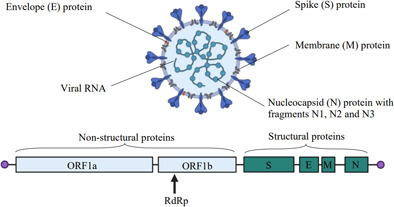
Illustration of the SARS‐CoV‐2 genomic regions targeted for amplification by various RT‐qPCR assays. Adapted from 43, “Genome Organization of SARS‐CoV” and “Genome Organization of SARS‐CoV‐2” templates from Biorender 13.

PCR uses a thermostable enzyme, typically *Taq Polymerase*, and probes/primers to target specific nucleic acid sequences for amplification 3, 39. For SARS‐CoV‐2 detection, RT‐qPCR has been the most frequently applied method 31, 40. Fig. 3 displays the structural composition and genomic regions of SARS‐CoV‐2 amplified during detection. Assay sensitivity, sample matrix, and reagent concentrations are the main factors affecting PCR amplification efficiency 41. An amplification efficiency greater than 100 % can result from experimental measurement error or presence of inhibitors that may co‐concentrate during concentration 41, 42.

Monitoring viruses in wastewater and wastewater‐impacted environments has the potential to aid in faster disease outbreak response and control 44. This study provides an overview on quantitative studies on SARS‐CoV‐2 occurrence in wastewater and feces using RT‐qPCR for detection, published online until April 9, 2021, including an analysis of the detection efficiency at different analytical stages during RT‐qPCR protocols, efficiency of the recovery by concentration, extraction, and amplification procedures.

Data presented in this review provides important information for future standardization of different analytical protocols enabling the assessment of available datasets for WBE and viral outbreak control decisions. However, there were few studies available on enveloped viruses that were not typically used as surrogates for viruses in wastewater, while detection methods for enteric viruses, of which the most are non‐enveloped, have been recently reviewed in aquatic environments 37.

## 3 Methodology

### 3.1 Data Search and Extraction

**Figure 4:**
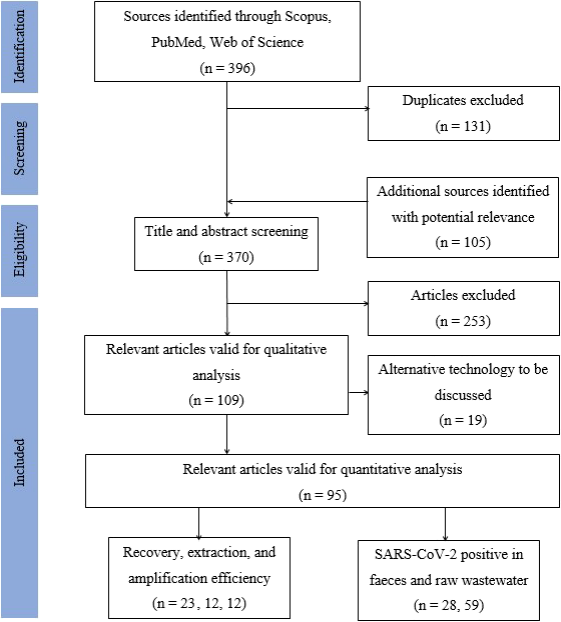
Flow diagram of literature screening and selection process (*n* = number of studies used for qualitative or quantitative analysis).

Data collection was conducted following PRISMA guidelines (Preferred Reporting Items for Systematic Review and Meta‐Analysis) 45. Fig. 4 details the literature screening and selection process. The following databases and descriptors were used: Scopus (search field = article title, abstract, keyword), PubMed (search field = all fields), and Web of Science (search field = topic). Inclusion and exclusion criteria are specified in Tab. 1. Qualitative information was extracted from virus species and strain/surrogates, type of sampling, i.e., grab or composite, in addition to concentration, extraction, detection method, and chemicals involved in the process. Quantitative data collated recovery by concentration, extraction, and amplification efficiency for experiments that followed RT‐qPCR protocols, and number of positive stool or raw wastewater samples collected for the specified viruses and surrogates. Box and whisker plots were prepared via the standardized percentile method of SigmaPlot 14.0 (Systat Software, San Jose, California). Graphical illustrations were made with Biorender 13. Forest plots were generated using data analysis in MedCalc for Windows, version 19.4 (MedCalc Software, Ostend, Belgium) 46.

**Table 1:** Inclusion and exclusion criteria relevant for data extraction.

Inclusion criteria	Exclusion criteria
– Specified quantitative data on SARS‐CoV‐2 detection from influent municipal/hospital raw sewage or wastewater obtained from spiking process control virus into sample or spiking untreated wastewater into distilled water.	– All other treated or disinfected effluents and other sample media (water/soil/air).
	– Efficiency values averaged across multiple samples (i.e., influent and effluent) or multiple process steps (i.e., concentration and extraction) and other data not necessary for the analysis.
– Medical data to confirm positive SARS‐CoV‐2 detection from hospitalized patients.	
– Peer‐reviewed English language reports only.	– Studies without specified process control virus, or efficiency calculation methods.
– Any location and sample/patient number.	
– RNA viruses.	– DNA viruses.
– All surrogate viruses for SARS‐CoV‐2.	– Clinical data regarding testing by respiratory or urine samples. Numerical data provided during surveillance of SARS‐CoV‐2 circulation.
– Considered electronegative membrane, PEG/Al(OH)_3_ precipitation, skimmed milk or direct flocculation, ultracentrifugation, and ultrafiltration.	
	– Efficiency values with too large deviation from the mean, justified by a threshold of ≤ 50 for the standard deviation.

### 3.2 Statistical Evaluation and Hypothesis Testing

Statistical analysis was conducted for significance of data regarding the ratio of positive testing of SARS‐CoV‐2 in feces and raw wastewater samples, and recovery and amplification efficiency variance between different concentration and sampling methods. Quantitative analytical data of SARS‐CoV‐2 in feces and raw wastewater samples was analyzed with random effect meta‐analysis proportion statistics in MedCalc. The random effects model was chosen to account for data limitations in relation to heterogeneity, the variations amongst the data, and potential publication bias, evaluated with the I^2^ statistic and Egger's tests, respectively. The weighted summary proportion is the pooled result of the individual studies, estimated with the Freeman‐Tukey transformation under the random effects model. The 95 % confidence interval (CI) was determined for each study to evaluate the uncertainty of the data.

Recovery and amplification efficiency data was analyzed using the following hypothesis testing 47:

- The null hypothesis states that the study findings do not suggest a significant difference between efficiencies estimated by different concentration and sampling methods, and the observed differences correspond with sampling or random error.
- The alternative hypothesis proposes that the study findings suggest effects that are not subjected to sampling or experimental error.

Statistical significance was analyzed in IBM SPSS Statistics for Windows, version 27 (IBM Corp, Armonk, New York) with the Independent‐Samples Mann‐Whitney U test based on suspected high heterogeneity. The exact level of significance, *p* ≤ 0.05, was indicated by the two‐tailed *p* value. Asymptomatic *p* values were yielded when groups with larger sample sizes were compared. Cohen's *d* and Mann Whitney *U* values and histograms are provided in the Supplementary Information.

### 3.3 Data Limitations

Available data of SARS‐CoV‐2 positive detection in feces samples had regional bias towards China. Variable reporting and sampling protocols and diverse virus concentration resulted by varied fecal shedding rates amongst infected patients are factors that constitute data heterogeneity. Reported recovery, extraction, and amplification efficiencies of several studies lacked statistically relevant information such as standard deviation, study internal variance, spiked virus, and sample numbers, while overall mean values were reported. If sample size was not available, the within‐study variance was removed from statistical efficiency evaluation. When possible, standard deviation was reported for individual data points of SARS‐CoV‐2 surrogates, due to the smaller dataset. Extraction efficiency data had to be excluded from analysis since the dataset was too small for statistical evaluation. Variability in wastewater characteristics, sample composition and treatment, and sample composition evaluated those average values amongst different environmental samples, such as influent and effluent, could not be included. Therefore, only raw wastewater was considered in the reported data. Publication bias was evident due to the small number of studies focusing on specific viruses or concentration methods. Some concentration methods did not provide a sufficient number of literature values comparison between enveloped and non‐enveloped viruses.

## 4 Detection of SARS‐CoV‐2 in Feces and Raw Wastewater

**Figure 5:**
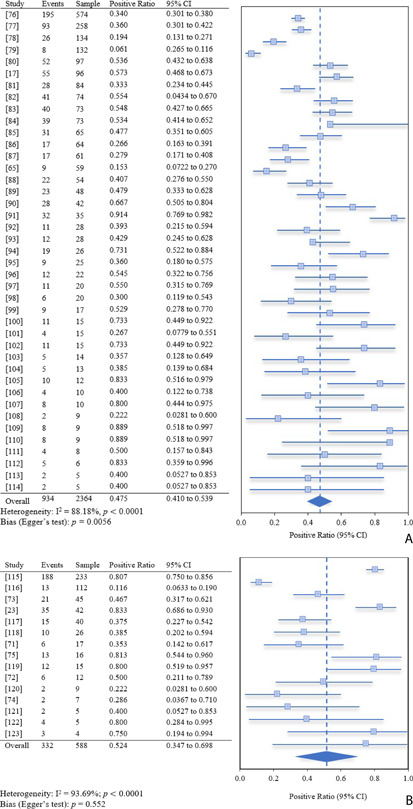
(A) Meta‐analysis forest plot of SARS‐CoV‐2 RNA positive detection ratio in fecal samples from patients with positive nasopharyngeal tests. The pooled estimate by random effect is represented by the diamond with 95 % confidence intervals. Event denotes the number of positive samples, whilst sample denotes the total number of collected samples. (B) Proportion meta‐analysis forest plot showing the positive rate of SARS‐CoV‐2 RNA detected in raw wastewater samples within communities of known virus outbreaks from nasopharyngeal testing. The pooled estimate by random effect is represented by the diamond with 95 % confidence intervals. Event denotes the number of positive samples, whilst sample denotes the total number of collected samples.

Forest plot Figs. 5A and 5B show the ratio of positive SARS‐CoV‐2 detection from medical studies from feces and measurements in raw wastewater samples of 41 and 15 studies, respectively. Graphical presentation of positive ratio, including 95 % CI, corresponds to numerical data presented on the left‐hand side tables. The dark blue diamond at the bottom of forest plots provides the weighted summary positive ratio across all studies. For overviews that include study location by country and the weighted percentage for each study within the analysis, see Supporting Information (SI) Tabs. S1 and S2.

The random effects estimate for the pooled positive detection rate was 47.5 % (95 % CI: 41.05–53.90 %) and 52.4 % (95 % CI: 34.7–69.8 %) for fecal and raw wastewater samples, respectively. Significant heterogeneity across the studies was evident in both figures (*p < *0.0001). Large CI and greater heterogeneity were found for raw wastewater samples, I^2^ = 93.69 %, and greater bias was evident in fecal samples, provided by low *p* value with the Egger's test (*p* = 0.0056). No evidence of publication bias was observed for studies collecting raw wastewater samples (*p* = 0.552). Larger study sizes carry greater weighting, while smaller sample sizes feature greater confidence intervals, and therefore incur less weight to the overall pooled estimate. Both datasets in the forest plots show an uneven distribution of sample size, due to studies with sample sizes < 20 falling to the right‐hand side of the mean, exhibiting a higher positive detection ratio, particularly in most fecal samples in Fig. 5A.

For fecal samples, 18 studies had 100 % detection rate 48, 49, 50, 51, 52, 53, 54, 55, 56, 57, 58, 59, 60, 61, 62, 63, 64, indicating significant presence of viral RNA in feces of infected patients. These studies are not displayed in Fig. 5A due to the forest plot representing the proportion of varied positive samples. A previous review on gastrointestinal occurrence of SARS‐CoV‐2 RNA load in feces reported that viral RNA was detected in 48.1 % of stool samples from 4243 infected patients collected across 60 studies 65. Gastrointestinal symptoms were present in 17.6 % of patients, and viral RNA concentration was greater in diarrhetic patients, 5.1 log_10_ copies mL^−1^, in comparison to 3.9 log_10_ copies mL^−1^ in the feces of patients without symptoms. This is in accordance with another study estimating that 16 % of 1141 confirmed patients experienced gastrointestinal symptoms 66.

Although there is significant detection of SARS‐CoV‐2 in wastewater samples, the diversity in viral shedding concentrations and rates must be considered to validate correlations. Fig. 5B expresses that a study had high prevalence, 80.7 %, in the greatest number of samples, 233 samples. Whilst four other studies had positive detection rates greater than or equal to 80 %, most of the studies had greater 95 % confidence intervals, indicating a low level of precision in the pooled estimate.

Additional information for studies summarized in Fig. 5B and other studies with 0 or 100 % RNA prevalence in samples are provided in Tab. 2, including study location, sampling method, targeted genome region for virus analysis, and positive RNA detection rate. Viral load in raw sewage has been investigated in many countries globally, with most studies conducting experiments with composite sampling. Variations in the ratio of positive samples may result from the different climates influencing virus survival and prevalence of infection at the sampled geographical locations. Temperature affects virus survivability, and evidence suggests coronaviruses inactivate faster at higher temperatures. For example, in tap water, the time required for the coronavirus titer to decrease by 99.9 % was 10 days in tap water at 23 °C, and > 100 days at 4 °C 67.

Another main factor affecting virus survivability is the composition of the wastewater, particularly in relation to the suspended solids and organic matter concentration due to increased electrostatic and hydrophobic adsorption 3, 33, 68. In addition, sewage hydraulic retention time, and transportation conditions of the obtained sample are likely to affect virus inactivation rates 69.

**Table 2:** SARS‐CoV‐2 abundance shown by positive RT‐qPCR detection in 2020/21 from the total number of raw wastewater samples.

Location		Sampling method	Genes in assay	Positive viral RNA detection rate	Ref.
Australia	Southeast Queensland	Composite	N	2/9	120
Brazil	Rio de Janeiro	Composite	N2	188/233	115
Czech Republic	Various WWTPs	Composite	N1, N2, N3	13/112	116
China	Wuhan	Grab	N, ORF1ab	0/4	27
England	South‐East	Composite	E, RdRp	2/5	121
Finland	Helsinki	Composite	E, N2	2/2	124
France	Paris	Composite	RdRp	3/3	125
Germany	North‐Rhine Westphalia	Composite	M, RdRp	9/9	29
India	Ahmedabad	Composite	N, ORF1ab, S	2/2	126
	Hyderabad Metropolitan City	Grab/composite	E, N, ORF1ab	9/9	127
	Jaipur	Grab	E, N, ORF1ab, RdRp, S	6/17	71
Iran	Tehran, Qom, Anzali	Composite	N, ORF1ab	12/12	128
Israel	Various WWTPs and hospitals	Composite	E	10/26	129
Japan	Ishikawa and Toyama	Grab	N2, N3	21/45	73
	Yamanashi	Grab	ORF1ab, N, S	0/5	130
Italy	Milan	Grab	E, N, ORF1ab	3/4	123
	Milan and Rome	Composite	ORF1ab, S	6/12	72
	Milan, Turin, Bologna	Composite	ORF1ab	15/40	117
	North of Italy, Stockholm, and Sweden	Grab	N	4/5	122
Netherlands	Various cities	Grab	E, N1, N2, N3	13/16	75
Spain	Murcia	Grab	N1, N2, N3	35/42	23
	Ourense	Composite	E, N, RdRp	5/5	131
	Valencia	Grab	N	12/15	119
Turkey	Istanbul	Grab	RdRp	5/7	132
USA	Montana	Composite	N1, N2	7/7	133
	Louisiana	Grab/composite	N1, N2	2/7	74
	Massachusetts	Composite	N1, N2, N3	10/10	134
	Michigan	Grab	N1	54/54	135

Different positive detection rates displayed in Tab. 2 may arise from varying assay specificity or inefficient primer design 70. Furthermore, greater positive results from wastewater samples were achieved by primers designed to target specific genes illustrated in Fig. 3, specifically E and N genes, compared to ORF1ab region, RdRp, and S gene 71, while others have found a higher positive detection ratio using ORF1ab compared to the S gene 72. Primers targeting specific regions of the N gene, N1, N2, and N3, have shown variable results with limited correlations available to assess analytical accuracy 23, 73, 74, 75.

## 5 Analysis of the Detection Efficiency at Various Process Stages

A list on all studies used for the analysis presented in this section is provided in SI, Tab. S3, including the publication date and location of study by country, studied viruses, sampling, pretreatment and concentration methods, and the respective recovery, extraction, and amplification efficiencies.

### 5.1 Addition of Process Control Viruses

Process control viruses are inoculated into the raw sample, concentrate or before amplification, to ensure quality control is measured at each stage of the detection method. These viruses, displayed in Tab. 3, can be surrogate viruses to SARS‐CoV‐2 or other infectious viruses that require investigation. Surrogate viruses, instead of actual human pathogens, are often used for experimental studies due to biosafety requirements and larger availability of the stock culture. SARS‐CoV‐2 surrogates are mostly enveloped viruses, such as MHV and bacteriophage Φ6, however, non‐enveloped viruses have been applied as a substitute, due to their common use as indicator quality control viruses in wastewater treatment. Process control viruses have importance in monitoring the process efficiency and the impact of inhibition. During data collection for this review it was found that many analytical protocols do not address inhibition mitigation strategies.

**Table 3:** Process control viruses utilized in the analysis of the detection procedure in raw wastewater.

Virus	Abbreviation	Efficiency	Ref.
*Enveloped surrogate viruses for SARS‐CoV‐2*			
Bacteriophage Φ6	Φ6	Recovery	136
Beta coronavirus	BCoV	Recovery	122
		Extraction	137
		Amplification	122
Bovine respiratory syncytial virus	BRSV	Recovery	115
Mouse hepatitis virus	MHV	Recovery	136, 137, 138
Porcine epidemic diarrhea virus	PEDV	Recovery	23
		Amplification	23
Transmissible gastroenteritis virus	TGEV	Extraction	116
*Non‐Enveloped surrogate viruses for SARS‐CoV‐2*			
Bacteriophage MS2	MS2	Recovery	136, 139, 140
		Amplification	140
Bacteriophage PP7	PP7	Recovery	115
F‐specific RNA phages (excluding MS2)	F‐phage	Recovery	73, 75
Mengovirus	MgV	Recovery	23, 119, 124, 141, 142
		Extraction	[143‐145]
		Amplification	23, 124
Murine norovirus	MNV	Extraction	73, 146, 147, 148
Pepper mild mottle virus	PMMoV	Recovery	137
		Extraction	149
		Amplification	122, 149
*Other Enveloped Virus*			
Influenza A	–	Recovery	150
		Extraction	150
Dengue virus	–	Extraction	75
Severe acute respiratory virus 2	SARS‐CoV‐2	Amplification	23, 29, 75, 122, 124, 127, 133, 134, 137, 140
*Other Non‐Enveloped Virus*			
Coxsackie B virus	–	Recovery	151
Echovirus 7	–	Recovery	152
Norovirus GII	–	Recovery	151, 153, 154, 155
		Amplification	124, 156
Poliovirus	–	Recovery	157
Rotavirus A	–	Recovery	158

### 5.2 Recovery and Amplification Efficiency Varied with Concentration Method

Accurate data collection for RNA viral load in wastewater is governed by the efficacy of concentration, extraction, and detection methods. Three types of efficiency were analyzed in this review which are (1) recovery: ratio of nucleic acid recovered and known amount spiked by the whole process control; (2) extraction: ratio of nucleic acid recovered and known amount spiked by the internal control; and (3) amplification: increase in target molecules amplified per PCR cycle.

**Figure 6:**
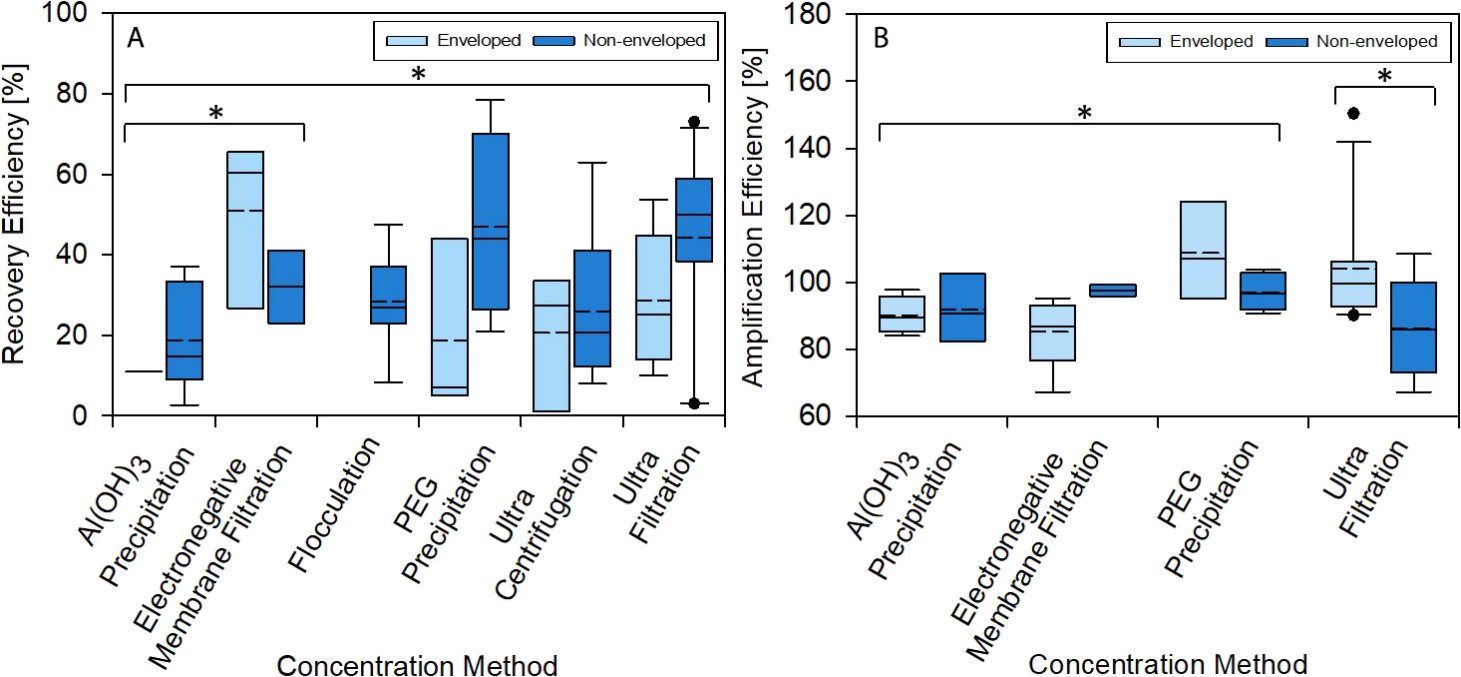
Recovery (A) and amplification (B) efficiencies for enveloped and non‐enveloped viruses for different concentration methods; * indicates statistical significance (*p* ≤ 0.05) between concentration methods. Box, whiskers, and circles denote the interquartile range, maximum, and minimum points, 5^th^ or 95^th^ percentiles, respectively. Solid and dotted lines within each box represent the median and mean datasets, respectively.

Figs. 6A and 6B illustrate recovery and amplification efficiencies for the enveloped and non‐enveloped viruses specified in Tab. 3 using different concentration methods. The figures show a collective dataset grouped into enveloped and non‐enveloped for each concentration method, necessary for determining which concentration method is currently the most widely used and most efficient. For recovery efficiency, 55 values were available from 23 separate studies. The data base consisted of enveloped/non‐enveloped: Al(OH)_3_ precipitation (*n* = 1 23 / *n* = 2 23, 142); electronegative membrane (*n* = 1 138 / *n* = 2 140, 157); PEG precipitation (*n* = 3 136, 137, 138 / *n* = 4 73, 136, 137, 152); ultracentrifugation (*n* = 3 115, 136, 138 / *n* = 5 115, 136, 139, 142, 158); ultrafiltration (*n* = 4 122, 136, 138, 150 / *n* = 7 75, 124, 136, 139, 141, 151, 158).

Viruses included in the analysis of recovery efficiency mainly consisted of enveloped and non‐enveloped SARS‐CoV‐2 surrogates, as well as non‐enveloped viruses more commonly found in wastewater. For amplification efficiency during RT‐qPCR detection, 41 values were available from 12 separate studies. The data base consisted of enveloped/non‐enveloped: Al(OH)_3_ precipitation (*n* = 1 23 / *n* = 1 23); electronegative membrane (*n* = 1 140 / *n* = 2 [23, 142); PEG precipitation (*n* = 2 134, 137 / *n* = 1 156); ultrafiltration (*n* = 6 29, 75, 122, 124, 127, 133 / *n* = 2 122, 124).

A larger dataset was obtained for concentration by ultrafiltration, whereas no data was obtained for concentration by flocculation or ultracentrifugation. Amplification efficiency data was mainly obtained from studies analyzing assay efficiency for SARS‐CoV‐2 detection but includes surrogate viruses and other viruses, specifically norovirus and PMMoV due to their role in wastewater environment circulation. Statistical analysis values, *p*, *U*, and Cohen's *d* [95 % CI] evaluated for the recovery efficiency of enveloped vs. non‐enveloped viruses and mean rank histograms for each concentration method are provided in the Supporting Information Tab. S4 and Fig. S1, respectively. Values for recovery efficiency varied with concentration method and mean rank histograms for the different concentration methods are provided in Tab. S5 and Fig. S2, respectively.

Average recovery efficiency of enveloped viruses was similar amongst concentration methods, particularly during PEG precipitation, ultracentrifugation, and ultrafiltration, ranging from 18 to 32 %. Despite this, there was no statistical significance (*p* > 0.05) observed between different concentration methods, possibly due to the small size of datasets. However, statistical significance was found for Al(OH)_3_ precipitation and electronegative membrane concentration (*p* = 0.03), and Al(OH)_3_ precipitation and ultrafiltration (*p* = 0.021) for all viruses. Low *p* values, indicating some statistical difference, were observed between ultrafiltration and ultracentrifugation (*p* = 0.061) and electronegative membrane and ultracentrifugation (*p* = 0.09). No data was available for enveloped viruses recovered by flocculation techniques, and one value, 11 %, was found for concentration by Al(OH)_3_ precipitation, indicating that these methods may not be widely applied for wastewater concentration prior to RT‐qPCR 23.

Larger datasets were available for non‐enveloped viruses, particularly for concentration by ultrafiltration. Recovery using electronegative membrane filtration was effective for enveloped MHV, whereas precipitation by PEG was more effective for recovering non‐enveloped viruses. MHV is an established process control, which could explain the better recovery when used for enveloped viruses. Low recovery for enveloped viruses during PEG precipitation could indicate susceptibility to organic chemicals used in the process that disrupt the virus lipid bilayer 136, 159. Low variability has previously been observed for concentration of waterborne pathogens in tap water by skimmed milk flocculation (SMF) 160. High variability was found for norovirus GII recovery, 118.7 ± 92.5 %, during concentration of raw wastewater by the same method 153. This value is not shown in Fig. 6A due to the high standard deviation beyond the inclusion criteria for this review. Large errors may stem from experimental issues, poor titration volumes, and presence of PCR inhibitors, such as humic acid, co‐concentrating with the method and leading to high apparent recovery efficiency of > 100 %.

Recovery of non‐enveloped viruses by SMF and Al(OH)_3_ precipitation was generally low, indicating these methods are less suitable for enveloped virus recovery. High organic concentration, large sample volume, and presence of inhibitors are factors leading to low recovery 161. No trends between processed sample volume and recovery of F‐specific RNA phages were observed previously 75. However, others have indicated that sample volume is a considerable factor affecting recovery efficiency, perhaps because of greater variation of components within the sample, resulting in poor sensitivity of the assay used for detection 31, 162.

The inoculum concentration of the process control virus also impacts the recovery efficiency as this is calculated from an initial and final concentration, which may not truly represent the proportion of virus in the original sample matrix. Source of viral stock, from stool or grown from a culture, could affect results, because there may be a greater concentration of free viral RNA in stool samples 151. Variable recovery efficiency of electronegative membranes may result from blocked membrane pores, particularly if no sample prefiltration is employed 163. High solids content of samples enhances virus retention on filter media, specifically for enveloped viruses 136. For SMF and electronegative membranes, the virus isoelectric point also influences the recovery efficiency 160, 164.

Ultracentrifugation and ultrafiltration generally do not require chemical reagents to modify the pH of the sample or influence electrostatic interactions. However, there are multiple factors such as the types and volume capacity of filters and centrifugal speeds that will affect recovery. Molecules greater than the molecular weight cut‐off will retentate during ultrafiltration techniques 163, increasing the likelihood of inhibitory effects during amplification. High variability of ultrafiltration may stem from the strength of virus adsorption by hydrophobic bonding or Van der Waal interactive forces 165.

During concentration, viral RNA adsorbed to the surface of solids accumulated in suspension would also be removed if these forces are not overcome with the method 166. Low recovery was observed for both virus types that were concentrated by ultracentrifugation. This could be the result of high centrifugal forces that potentially inactivate and disrupt the viral structure due to mechanical stress 167. Despite this, fragmented viral nucleic acid has been detected by RT‐qPCR 168. More nucleic acid fragments may lead to an overestimation of the actual viral concentration, and this reinforces that the detection process requires improvements for greater accuracy.

Fig. 6B indicates that amplification efficiency is within a range of approximately 70–140 %, while the desired range of efficiency for PCR amplification is between 90–110 % 169. Although some experiments exceeded this range, the mean and median remain close to 100 %. Statistical significance (*p* = 0.029) was established between enveloped and non‐enveloped virus amplification after ultrafiltration. No statistical significance (*p* > 0.05) was established between enveloped and non‐enveloped viruses for other methods, despite being low (*p* = 0.095) for electronegative membrane filtration. When comparing concentration methods for all viruses, statistical significance (*p* = 0.023) was observed between Al(OH)_3_ and PEG precipitation.

Other methods had no statistical significance (*p* > 0.05), however, the *p* value was low (*p* = 0.071) between electronegative membrane filtration and PEG precipitation. Statistical analysis values, *p*, *U*, and Cohen's *d* [95 % CI], for amplification efficiency of enveloped vs. non‐enveloped viruses and mean rank histograms for each concentration method are provided in SI Tab. S6 and Fig. S3, respectively. Amplification efficiency variations and mean rank histograms for the different concentration methods are provided in Tab. S7 and Fig. S4, respectively.

### 5.3 Extraction Efficiency Varied with Concentration Method

Tab. 4 compares different extraction methods for both enveloped and non‐enveloped viruses. Extraction efficiencies are shown in table format due to the large heterogeneity in the small dataset for the SARS‐CoV‐2 surrogates and other enveloped and non‐enveloped viruses identified as process control viruses. Note that concentration methods, structure of virus, and low titers of virus inoculum are factors responsible for variable efficiency throughout the process 144. Differences in protocols through use of different concentrations of chemical reagents or buffer solutions could explain the variance in recovery efficiency observed in Fig. 6A and Tab. 4. One study recovered the virus by direct RNA extraction without concentration 147, which may be a faster and cost‐effective alternative 170. Nevertheless, large variability indicates the requirement for standardized protocols of virus detection in water matrices.

**Table 4:** Extraction efficiencies of various enveloped and non‐enveloped viruses.

Structure	Concentration method	Extraction kit	Species	Efficiency	Ref.
Enveloped	Direct flocculation (with beef extract)	NucliSENS miniMAG system (Biomerieux)	TGEV	35.53 ± 13.04 %	116
	PEG precipitation	AllPrep PowerViral DNA/RNA kit (Qiagen)	BCoV	26 %	137
	Ultrafiltration	RNeasy PowerMicrobiome Kit (Qiagen)	Dengue	30.4 ± 22.3 %	75
		NucliSENS kit (Biomerieux)	Influenza A	100 % and 92 %	150
Non‐enveloped	Electronegative membrane	ZR Viral DNA–RNA Kit (Zymo Research)	MNV	90.4 ± 34.4 %, 108.8 ± 44.4 %	146
		QIAamp		114 %	148
		Viral RNA Mini Kit			
		QIAmp Viral RNA Mini Kit (Qiagen)			
		QIAmp MinElute Virus Spin Kit (Qiagen)	PMMoV	32.4 %	149
	None	NucliSENS easyMAG system (Biomerieux)	MNV	> 35 %	147
	PEG precipitation	NucliSENS kit (Biomerieux)	MgV	10, 11, 12, 13 %	143
		Phenol‐chloroform‐water and chloroform‐isoamyl alcohol		117 ± 96 %	144
		QIAmp Viral RNA Mini Kit (Qiagen)	MNV	83± 2 %	73
	Ultracentrifugation	NucleoSpin RNA virus kit (Macherey‐Nagel)		8.835 %	145

### 5.4 Recovery Efficiency Varied with Sampling Techniques and Additives

**Figure 7:**
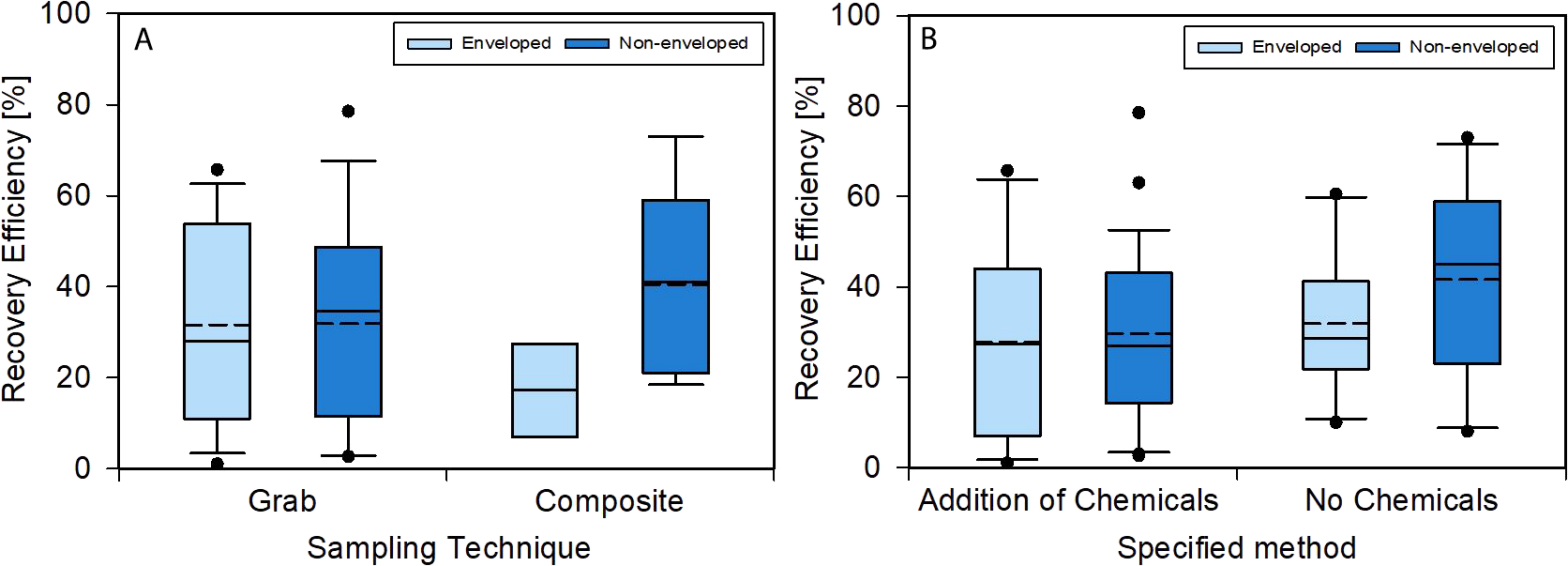
Recovery efficiencies for enveloped and non‐enveloped viruses under different sampling protocol (A) and chemical methods (B). Box, whiskers, and circles denote the interquartile range, maximum, and minimum points, 5^th^ or 95^th^ percentiles, respectively. Solid and dotted lines within each box represent the median and mean datasets, respectively.

Fig. 7A provides a comparison on recovery efficiency for grab and composite sampling, whilst Fig. 7B shows the effect of chemical additives used during the recovery process for enveloped and non‐enveloped viruses. For data in Fig. 7A, a total number of 31 and 9 values for enveloped and non‐enveloped viruses were available for grab and composite sampling, respectively. Amongst sampling techniques and virus structure, no statistical significance (*p > *0.05) was observed, most likely due to the heterogeneity within the small database. Grab sample results are more likely to be affected by short‐term peaks of viral RNA in raw sewage or wastewater, while composite samples might provide more accurate representations of average concentration across a longer time period 73, 133. Composite sampling recovery was lower for enveloped viruses, while only two datasets were available 115, 137.

Several outliers were found for recovery after grab sampling, as illustrated by circles in Figs. 7A and 7B, indicating some variation, perhaps due to different sampling times and locations 162. Most studies reporting SARS‐CoV‐2 in raw wastewater were composite samples as indicated in Tab. 2, while higher virus concentrations might be found in grab samples due to greater loads amongst countries with variable infection rates and outbreaks 73. Statistical analysis values, *p*, *U*, and Cohen's *d* [95 % CI], for recovery efficiency compared for sampling type and mean rank histograms for different viruses and sampling types are provided in SI Tab. S8 and Fig. S5, respectively. Values for recovery efficiency with use of chemical additives and mean rank histograms for different viruses and specified methods are provided in Tab. S9 and Fig. S6, respectively.

Chemical additives, such as cationic adsorption inhibitors, pH buffers, surfactants, disinfectants, inhibition reductants, and DNA/RNA stabilizers used during sampling processing, had only a minor effect on recovery of enveloped viruses, by 4.4 %, with greater recovery observed without additives, whereas recovery efficiency for non‐enveloped viruses was approximately 12.0 % lower with additives, as illustrated in Fig. 7B. Despite this, no statistical significance (*p* > 0.05) was observed. Enveloped viruses have greater susceptibility to various additives due to the sensitivity of the lipid bilayer 171. However, some concentration methods, such as organic flocculation, require strongly acidic conditions that consequently inactivate the infectious virus by disruption of lipid envelopes or integral structure of capsids and nucleic acids 165. In some cases, chemical additives act inhibitory, e.g., proteins present in beef extract 165, and excess salts or surfactants 172. Beyond chemical additives, pasteurization heat treatment increases virus inactivation in wastewater 136 and may induce RNA fragmentation, which can affect the amplification process 173. Evaluation of specific virus survival characteristics to particular chemical additives would provide insight into the explanation of variations in recovery efficiency.

### 5.5 Recovery Efficiency for Surrogate Viruses

**Figure 8:**
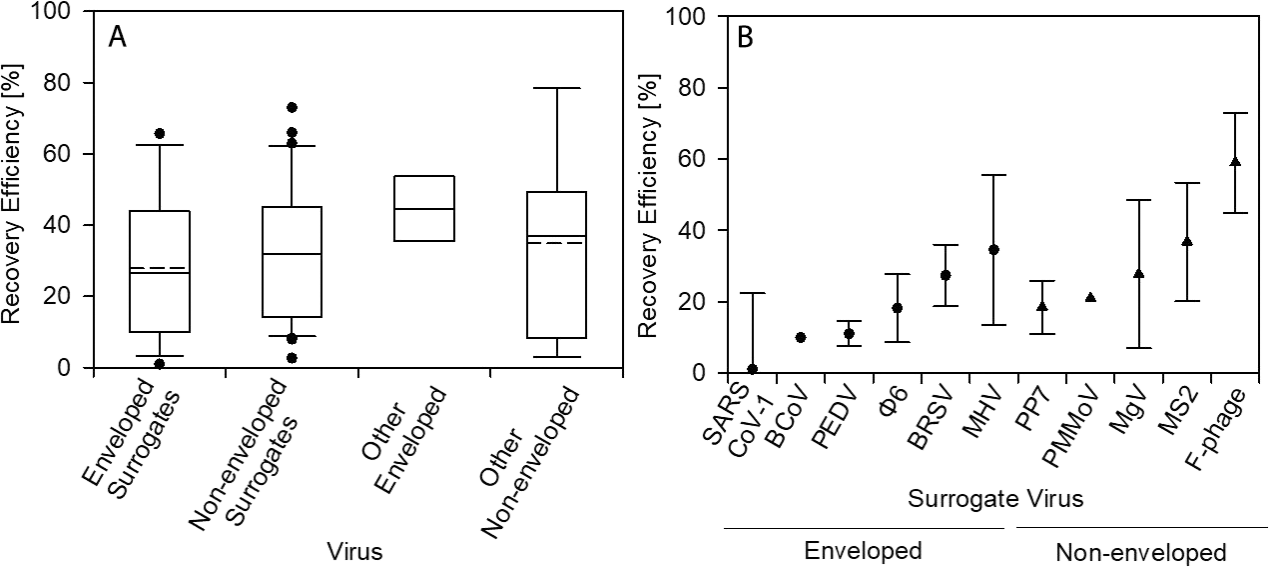
Recovery efficiency of enveloped and non‐enveloped surrogates and non‐surrogate viruses inoculated into raw wastewater samples for SARS‐CoV‐2 analysis (A). Recovery efficiency for different species of enveloped and non‐enveloped surrogates for SARS‐CoV‐2 analysis in raw wastewater (B). Error bars represent standard deviation of mean recovery efficiency. Box, whiskers, and circles denote the interquartile range, maximum, and minimum points, 5^th^ or 95^th^ percentiles, respectively. Solid and dotted lines within each box represent the median and mean datasets, respectively.

Fig. 8A illustrates the recovery efficiency for different surrogates and other virus species used for SARS‐CoV‐2 analysis in raw wastewater across enveloped and non‐enveloped viruses, whilst Fig. 8B individually displays the mean recovery efficiency for SARS‐CoV‐2 surrogates. Fig. 8A shows that the mean recovery efficiency for non‐enveloped viruses was slightly greater than for enveloped and non‐enveloped surrogate viruses, although not statistically significant (*p* > 0.05). The minor difference may reflect higher robustness of non‐enveloped viruses and their predominant transmission via the fecal‐oral route, including through wastewater. Other enveloped viruses were represented by influenza A, although only reported by one study 150. Statistical analysis values, *p*, *U*, and Cohen's *d* [95 % CI], for surrogate viruses and mean rank histograms for the different virus groups are provided in SI Tab. S10 and Fig. S7, respectively.

Fig. 8B illustrates error bars representing the standard deviation from the mean recovery efficiency, which was extracted for surrogates provided by one study. No standard deviation was given for PMMoV and BCoV, and this study stated that BCoV as a surrogate did not offer meaningful data to interpret results due to low recovery efficiency 122. Greater deviation was observed as recovery efficiency increased, most likely due to larger datasets representing more differences between experimental conditions. MHV, bacteriophage MS2, and other filamentous bacteriophages have greater mean recovery efficiencies, i.e., 32.0, 36.8, and 59.0 %, respectively. Recovery efficiency for surrogate viruses may not accurately represent SARS‐CoV‐2 behaviour 73. Surrogate viruses exhibit different structures and genome compositions than target viruses based on their specific life cycle. Given high structural similarity, i.e., 82 %, between SARS‐CoV‐1 and SARS‐CoV‐2, recoveries could be assumed similar 75. SARS‐CoV‐1 recovery in raw sewage by electropositive membrane filtration was 1.02–21.4 %, with estimated infectivity for up to two days 174. Low recovery suggests poor detection efficacy and virus survival in sewage.

## 6 Conclusions

This review provides a timely intermediate analysis of the quickly growing database on SARS‐CoV‐2 detection in feces and wastewater, offering quantitative information on RT‐qPCR detection of non‐enveloped and enveloped viruses in wastewater. Whilst literature discusses the potential of WBE as a viable method of detecting positive SARS‐CoV‐2 in pooled samples, the challenge of successful integration remains, as well as the existence of wastewater matrix inhibitors that result in inaccuracies present in the collected data from the RT‐qPCR process. By comparing the current statistics on positive detection by different methodological approaches in the RT‐qPCR process, research can be directed into standardizing the most frequently used methods to be used in future pandemics.

SARS‐CoV‐2 RNA has been detected in fecal and raw wastewater environments, with mean positive rates of 47.5 % (95 % CI: 41.05–53.90 %) and 52.4 % (95 % CI: 34.7–69.8 %) respectively, while 18 studies were excluded from the analysis due to positive detection rates of 100 %. Recovery efficiency values were available for SARS‐CoV‐2 detection and its surrogates as well as norovirus, that typically ranged between 18–32 % for enveloped viruses by PEG precipitation, ultracentrifugation, and ultrafiltration, without significant difference between methods and virus types. Despite this, statistical significance of recovery efficiencies between Al(OH)_3_ precipitation and electronegative membrane (*p* = 0.03), Al(OH)_3_ precipitation and ultrafiltration (*p* = 0.021) was observed. Amplification efficiency was analysed, and available literature data displayed mean and median values close to 100 %, for both enveloped and non‐enveloped viruses, while the overall range of 70–140 % was higher than the desired range of 90–110 %.

Statistical significance of amplification efficiencies amongst enveloped and non‐enveloped virus amplification after ultrafiltration (*p* = 0.029), and amplification after Al(OH)_3_ and PEG precipitation (*p* = 0.023) was established. Various factors likely affecting recovery rates of specific viruses and concentration methods included sampling methods, safety and handling requirements of infectious viruses, chemical inactivation of viruses, fluctuations of virus concentrations, solid‐virus attachment, and the presence of PCR inhibitors. Current efforts on SARS‐CoV‐2 detection may lead to developing standardized methodological approaches for enveloped virus detection in wastewater for more accurate detection and monitoring of a greater variety of public health relevant viruses, including improved viral diagnostic testing for WBE and faster responses to mitigate virus outbreaks.

## Supporting Information

Supporting Information for this article can be found under DOI: https://doi.org/10.1002/cben.202100039.

## Conflicts of Interest

The authors declare no conflict of interest.

## Abbreviations

Al(OH)_3_
: aluminium hydroxide
BCoV: beta coronavirus
BRSV: bovine respiratory syncytial virus
CI: confidence interval
CoV: coronavirus
COVID‐19: coronavirus disease 19
DNA: deoxyribonucleic acid
E: envelope protein
F‐phage: filamentous bacteriophage
M: membrane protein
MgV: mengovirus
MHV: mouse hepatitis virus
MNV: murine norovirus
MPC: molecular process control
N: nucleocapsid protein
ORF1ab: open reading frame 1a and 1b
PCR: polymerase chain reaction
PEDV: porcine epidemic diarrhea virus
PEG: polyethylene glycol
PMMoV: pepper mottle mild virus
PRISMA: preferred reporting items for systematic review and meta‐analysis
RNA: ribonucleic acid
RT‐qPCR: quantitative real‐time reverse transcription polymerase chain reaction
S: spike protein
SARS‐CoV‐1: severe acute respiratory syndrome coronavirus 1
SARS‐CoV‐2: severe acute respiratory syndrome coronavirus 2
SMF: skimmed milk flocculation
TGEV: transmissible gastroenteritis virus
WBE: wastewater‐based epidemiology
WPC: whole process control
WWTP: wastewater treatment plant

## Supporting information

Supplementary Information
