# Supplementary material for: Review and Meta‐Analysis: SARS‐CoV‐2 and Enveloped Virus Detection in Feces and Wastewater
Source: Chembioeng Reviews. 2022 Mar 3;9(2):129–45. doi: 10.1002/cben.202100039 (PMC9083821; doi:10.1002/cben.202100039)
Supplement: Supplementary file 1 — Supplementary Information [file CBEN-9-129-s001.pdf]

Supporting Information

Review and Meta-Analysis: SARS-CoV-2 and Enveloped Virus Detection in Feces and Wastewater

Charlotte Twigg, Jannis Wenk*

DOI: 10.1002/cben.202100039

Correspondence: Jannis Wenk (jhw46@bath.ac.uk), University of Bath, Department of Chemical Engineering and Water Innovation and Research Centre (WIRC@Bath), Claverton Down, Bath, Somerset, BA2 7AY, United Kingdom.

WILEY-VCH

Table S1. Overview on studies providing SARS-CoV-2 RNA positive detection ratio in fecal samples from patients with positive nasopharyngeal tests. Data used to prepare Figure 6A in main part of study.

Publication			Quantitative Data				
Reference	Year	Origin	Positive Samples	Total Samples	Proportion	95% CI	Weight [%]
[1]	2020	China	195	574	0.340	0.301 to 0.380	3.15
[2]	2020	China	93	258	0.360	0.301 to 0.422	3.1
[3]	2020	Italy	26	134	0.194	0.131 to 0.271	3.02
[4]	2020	Spain	8	132	0.061	0.265 to 0.116	3.02
[5]	2020	China	52	97	0.536	0.432 to 0.638	2.96
[6]	2020	China	55	96	0.573	0.468 to 0.673	2.96
[7]	2020	China	28	84	0.333	0.234 to 0.445	2.93
[8]	2020	China	41	74	0.554	0.0434 to 0.670	2.9
[9]	2020	China	40	73	0.548	0.427 to 0.665	2.9
[10]	2020	China	39	73	0.534	0.414 to 0.652	2.9
[11]	2020	China	31	65	0.477	0.351 to 0.605	2.87
[12]	2020	China	17	64	0.266	0.163 to 0.391	2.86
[13]	2020	China	17	61	0.279	0.171 to 0.408	2.85
[14]	2020	China	9	59	0.153	0.0722 to 0.270	2.84
[15]	2020	China	22	54	0.407	0.276 to 0.550	2.81
[16]	2020	USA	23	48	0.479	0.333 to 0.628	2.77
[17]	2020	China	28	42	0.667	0.505 to 0.804	2.72
[18]	2020	China	32	35	0.914	0.769 to 0.982	2.65
[19]	2020	China	11	28	0.393	0.215 to 0.594	2.54
[20]	2020	China	12	28	0.429	0.245 to 0.628	2.54
[21]	2020	Netherlands	19	26	0.731	0.522 to 0.884	2.5
[22]	2020	China	9	25	0.360	0.180 to 0.575	2.48
[23]	2020	China	12	22	0.545	0.322 to 0.756	2.41
[24]	2020	China	11	20	0.550	0.315 to 0.769	2.36
[25]	2020	China	6	20	0.300	0.119 to 0.543	2.36
[26]	2020	China	9	17	0.529	0.278 to 0.770	2.26
[27]	2020	Italy	11	15	0.733	0.449 to 0.922	2.18
[28]	2020	China	4	15	0.267	0.779 to 0.551	2.18
[29]	2020	China	11	15	0.733	0.449 to 0.922	2.18
[30]	2020	China	5	14	0.357	0.128 to 0.649	2.14
[31]	2020	China	5	13	0.385	0.139 to 0.684	2.09
[32]	2020	China	10	12	0.833	0.516 to 0.979	2.04
[33]	2020	China	4	10	0.400	0.122 to 0.738	1.91
[34]	2020	China	8	10	0.800	0.444 to 0.975	1.91
[35]	2020	China	2	9	0.222	0.0281 to 0.600	1.84
[36]	2020	Germany	8	9	0.889	0.518 to 0.997	1.84
[37]	2020	China	8	9	0.889	0.518 to 0.997	1.84
[38]	2020	Singapore	4	8	0.500	0.157 to 0.843	1.75
[39]	2020	China	5	6	0.833	0.359 to 0.996	1.55
[40]	2020	France	2	5	0.400	0.0527 to 0.853	1.43
[41]	2021	China	2	5	0.400	0.0527 to 0.853	1.43
Overall			934	2364	0.475	0.410 to 0.539	100

WILEY-VCH

Table S2. Overview on studies providing positive rate of SARS-CoV-2 RNA detected in raw wastewater samples within communities of known virus outbreaks from nasopharyngeal testing. Data used to prepare Figure 6B in main part of study.

Publication			Quantitative Data				
Reference	Year	Origin	Positive Samples	Total Samples	Proportion	95% CI	Weight [%]
[42]	2021	Brazil	188	233	0.807	0.750 to 0.856	7.65
[43]	2020	Czech Republic	13	112	0.116	0.0633 to 0.190	7.57
[44]	2021	Japan	21	45	0.467	0.317 to 0.621	7.35
[45]	2020	Spain	35	42	0.833	0.686 to 0.930	7.33
[46]	2021	Italy	15	40	0.375	0.227 to 0.542	7.31
[47]	2020	Israel	10	26	0.385	0.202 to 0.594	7.11
[48]	2020	India	6	17	0.353	0.142 to 0.617	6.85
[49]	2020	Netherlands	13	16	0.813	0.544 to 0.960	6.8
[50]	2020	Spain	12	15	0.800	0.519 to 0.957	6.75
[51]	2020	Italy	6	12	0.500	0.211 to 0.789	6.56
[52]	2020	Australia	2	9	0.222	0.0281 to 0.600	6.28
[53]	2020	USA	2	7	0.286	0.0367 to 0.710	6
[54]	2020	England	2	5	0.400	0.0527 to 0.853	5.58
[55]	2021	Sweden	4	5	0.800	0.284 to 0.995	5.58
[56]	2020	Italy	3	4	0.750	0.194 to 0.994	5.29
Overall			332	588	0.524	0.347 to 0.698	100

WILEY-VCH

Table S3. Overview on all studies used in Section 5 *Analysis of the Detection Efficiency at Various Process Stages* in the main study.

Publication			Virus Classification			Sampling	Pre-Treatment		Process prior to RT-qPCR detection	Quantitative Data		
Reference	Year	Origin	Structure	Family	Species	Type	Pasteurized	Chemical Reagents	Concentration Method	Recovery Efficiency [%]	Extraction Efficiency [%]	Amplification Efficiency [%]
[57]	2016	USA		Cystoviridae	Bacteriophage Φ6	Grab	No	-	Ultrafiltration	18.20		
[55]	2021	Sweden		Coronaviridae	Beta Coronavirus	Grab	No	-		10.00		99.50
[58]	2021	USA				Composite	No	Yes	PEG precipitation		26.00	
[42]	2021	Brazil		Paramyxoviridae	Bovine Respiratory Syndrome Virus	Composite	Yes	Glycine buffer, PBS	Ultracentrifugation	27.40		
[49]	2020	Nether-lands		Flaviviridae	Dengue Virus	Composite	No	-			30.40	
[59]	2011	Nether-lands		Orthomyxoviridae	Influenza A	Grab	No	-		53.80	100.00	
					Influenza A H1N1 2009	Grab	No	-	Ultrafiltration	35.50	92.00	
[57]	2016	USA				Grab	No	-		25.10		
						Grab	No	PBS		56.00		
						Grab	No	PBS		28.00		
[60]	2020	Australia				Grab	No	HCL		26.70		
			Enveloped			Grab	No	Neutral pH	Electronegative membrane	60.50		
					Mouse Hepatitis Virus	Grab	No	MgCl2		65.70		
						Grab	No	PEG, NaCl, beef extract in glycine, HCl		44.00		
[57]	2016	USA		Coronaviridae		Grab	No	PEG, NaCl, PBS (pH: 7.4)	PEG precipitation	5.00		
						Grab	No	Glycine buffer, PBS, Beef extract, NaCl		1.00		
[60]	2020	Australia				Grab	No	Glycine buffer, PBS	Ultracentrifugation	33.50		
[58]	2021	USA				Composite	No	Yes	PEG precipitation	7.00		
[45]	2020	Spain			Porcine Epidemic Diarrhoea Virus	Grab	No	AlCl3 to adjust pH to 6.0	$Al(OH)_3$ precipitation	11.00		89.47
[55]	2021	Sweden				Grab	No	-				90.20 (N)
[49]	2020	Nether-lands			Severe Acute Respiratory Virus 2	Composite	No	-	Ultrafiltration			98.50 ± 1.30, 94.30 ± 5.20, and 94.30 ± 4.10 for N1, N2, and N3

[61]	2021	India				Both	Heat treatment	NaOCl and disinfection with ethanol				106.55, 105.59, 103.01 (E, N, ORF1ab gene)
[62]	2021	Germany				Composite	No	Deionized water				90.80 (M-gene)
[63]	2020	USA				Composite	No	-				150.36 (N1)
						Composite	No	-				129.45 (N2)
[64]	2021	Finland				Composite	No	None	Ultrafiltration			99.50 (E), 91.00 (N2)
[65]	2020	USA				Composite	Yes	PEG, NaCl, TRIzol	PEG precipitation			107.10 (N1)
						Composite	Yes	PEG, NaCl, TRIzol				95.23 (N2)
						Composite	Yes	PEG, NaCl, TRIzol				124.12 (N3)
[45]	2020	Spain				Grab	No	AlCl3 to adjust pH to 6.0	$Al(OH)_3$ precipitation			97.74 (N1), 84.03 (N2), 89.55 (N3)
[66]	2021	Germany				Composite	No	Not specified	Electronegative membrane			95.32 ± 9.09 for N, 91.09 ± 13.84 for S, 86.75 ± 1.80 for Orf1ab
[58]	2021	USA				Composite	No	Yes	PEG precipitation			89.60 - 98.50 (median: 94.05 N1), 88.10 - 96.20 (median: 92.15 N2)
[67]	2005	China			Severe Acute Respiratory Virus 1	Composite	No	Na2CO3, AlCl3 , Silica gel to form Al(OH)3 precipitate	Positive charged filter media	1.02 (range 0 - 21.4%)		
[68]	2020	Czech Republic			Transmissible Gastroenteritis Virus	Composite	No	Beef extract, glycine buffer, HCl, PBS	Direct Flocculation		35.53	
[57]	2016	USA	Non-enveloped	Fiersviridae	Bacteriophage MS2	Grab	No	-	Ultrafiltration	55.60		
[69]	2020	Spain				Not specified		-		50.00		
										43.00		
										66.00		
										45.00		
[57]	2016	USA				Grab	No	PEG, NaCl, PBS (pH: 7.4)	PEG precipitation	43.10		
						Grab	No	Glycine buffer, PBS, Beef extract, NaCl	Ultracentrifugation	63.00		
[69]	2020	Spain				Not specified		-		8.00		
										23.00		

										23.00		
										12.00		
[66]	2021	Germany				Composite	No	Yes	Electronegative membrane	41.14		95.92
[69]	2020	Spain				Not specified		Yes	Skimmed Milk Flocculation	23.00		
										37.00		
										32.00		
										24.00		
[42]	2021	Brazil		Leviviridae	Bacteriophage PP7	Composite	Yes	Glycine buffer, PBS	Ultracentrifugation	18.50		
[70]	2016	Canada		Picornaviridae	Coxsackie B	Grab	No	PBS	Ultrafiltration	median: 50.00, range: 4.00 - 75.00		
[71]	2012	Morocco			Echovirus 7	Grab	Yes	PEG, NaCl, chloroform, PBS	PEG precipitation	78.50 (Virions), 10.33 (RNA)		
[49]	2020	Nether-lands		Leviviridae	F-specific RNA phages (excluding MS2)	Composite	No	-	Ultrafiltration	73.00		
[44]	2021	Japan				Grab	No	PEG, NaCl, phosphate buffer	PEG precipitation	45.00		
[72]	2018	UK		Picornaviridae	Mengovirus	Grab	No	Virkon® solution (Lanxess, Germany), beef extract and NaNO3, PEG	Tangential Flow Ultrafiltration (TFUF) and PEG Precipitation for sediments	38.23		
[64]	2021	Finland				Composite	No	None	Ultrafiltration	59.00		median: 87.50
[45]	2020	Spain				Grab	No	AlCl3 to adjust pH to 6.0	$Al(OH)_3$ precipitation	11.00		82.48
[73]	2016	France				Grab	No	PEG	PEG precipitation		10.00, 11.00, 12.00, 13.00	
[74]	2008	USA				Grab	No	PEG, proteinase K to extract acid, chloroform water, cetyltrimethylammonium bromide, NaCl, ethanol, Rnase inhibitor, glycine buffer			117 ± 96	
[75]	2019	Spain				Grab	No	PBS	Ultracentrifugation	13.09		
						Grab	No	AlCl3 to adjust pH to 6.0, beef extract, PBS	$Al(OH)_3$ precipitation	2.67		
						Grab	No			15.33		
						Grab	No			32.25		
						Grab	No	PMAXX pre-treatment, AlCl3 to adjust pH to 6.0, beef extract, PBS		14.29		
						Grab	No			37.08		

[76]	2018	Spain				Not specified	No	PMAxx pre-treatment, glycine buffer, PBS	Ultracentrifugation		4.37-13.30 (mean: 8.84)	
[50]	2020	Spain				Grab	No	AlCl3 to form Al(OH)3 precipitate, PBS	Direct Al driven Flocculation	8.28		
[44]	2021	Japan		Caliciviridae	Murine Norovirus	Grab	No	PEG, NaCl, phosphate buffer	PEG precipitation		83.00	
[77]	2016	USA				Both	No	MgCl2, H2SO4, NaOH, Tris-EDTA buffer	Electronegative membrane		90.40, 108.80	
[78]	2013	Italy				Grab	No	Glycine, chloroform	Direct RNA extraction		35.00	
[79]	2013	Japan				Composite	No	MgCl2, H2SO4, NaOH, Tris-EDTA buffer	Electronegative membrane		114.00 (99.00 – 130.00)	
[80]	2018	Japan			Norovirus GII	Composite	No	PEG, NaCl, deionized water, TE buffer	PEG precipitation			99.20
						Composite	No					103.80
						Composite	No					102.50
						Composite	No					90.80
[70]	2016	Canada				Grab	no	PBS	Ultrafiltration	median: 3.00, range: 1.00 - 8.00		
[64]	2021	Finland				Composite	No	None	Ultrafiltration			108.50
[81]	2019	Brazil				Grab	No	Glycine buffer, HCl, PBS	Skimmed Milk Flocculation	118.70		
[82]	2004	UK				Not specified	No	Beef extract, Na2HPO4 buffer pH 7	Flocculation with HCL at pH 3.5	27.00		
[83]	2013	Spain				Composite	No	Phosphate buffer, glycine buffer, HCl	Skimmed Milk Flocculation	range: 45.00 -50.00		
[55]	2021	Sweden		Virgaviridae	Pepper Mottle Mild Virus	Grab	No	-	Ultrafiltration			91.50, 78.70
[55]	2021	Sweden				Grab	No	MgCl2	Electronegative membrane			67.30, 85.9 0
[84]	2016	USA				Grab	No	Acetic acid			32.40	99.38
[58]	2021	USA				Composite	No	Yes	PEG precipitation	21.00		
[85]	2008	Japan		Picornaviridae	Poliovirus	Composite	No	MgCl2, H2SO4, NaOH, Tris-EDTA buffer	Electronegative membrane	23.00		
[86]	2010	Brazil		Reoviridae	Rotavirus A	Not specified	No	Acid rinse step, MgCl2. H2SO4, NaOH, TE buffer, bleach solution and deionized H20	Ultrafiltration	3.50		
						Not specified	No	Glycine buffer, PBS	Ultracentrifugation	47.00		

Recovery Efficiency: Enveloped vs non-enveloped for each concentration method

Table S4. Statistical values for recovery efficiency of enveloped and non-enveloped virus groups compared for the different concentration methods.

Enveloped vs Non-enveloped Virus Compared for Concentration Methods	*p* value	U	Cohen's d (95% CI)
All methods	0.629	349.500	0.156 (-0.417 to 0.728)
$Al(OH)_3$ Precipitation	0.571	4.500	0.589 (-1.585 to 2.708)
Electronegative Membrane Filtration	0.400	1.000	1.005 (-1.006 to 2.886)
PEG Precipitation	0.229	10.000	1.226 (-0.490 to 2.848)
Ultracentrifugation	1.000	12.000	0.283 (-1.058 to 1.609)
Ultrafiltration	0.246	52.000	0.570 (-0.406 to 1.529)

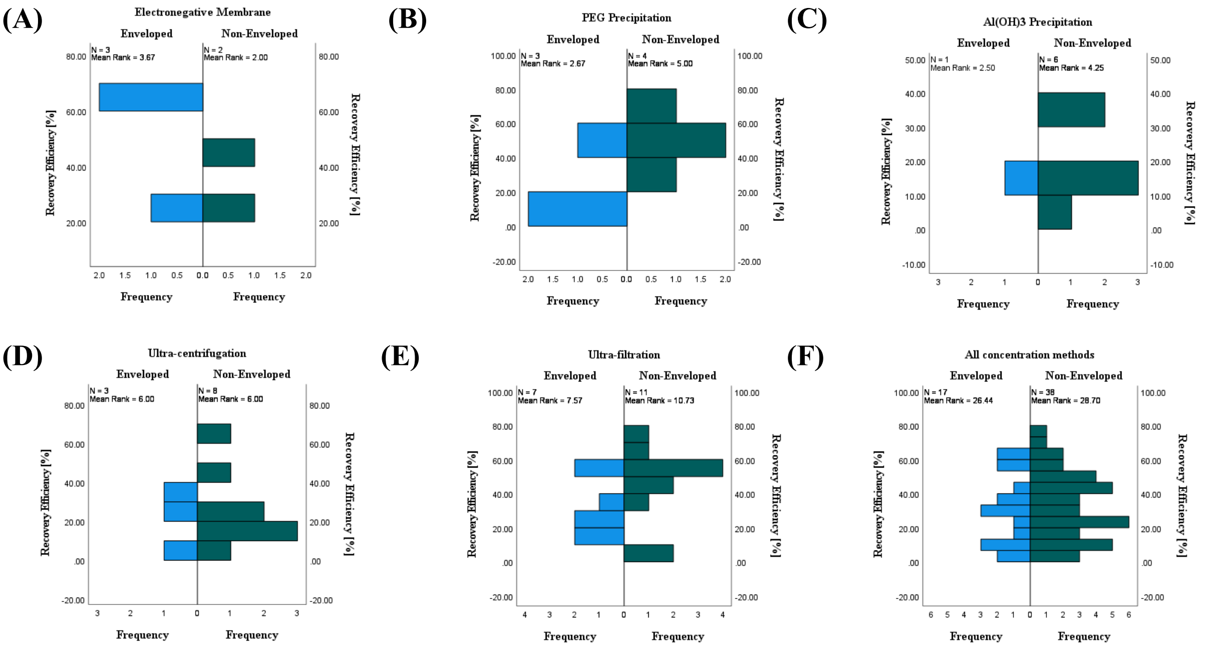

Figure S1. Histograms displaying the mean rank value for recovery efficiency of enveloped and non-enveloped virus groups compared for the different concentration methods: (A) Electronegative membrane filtration, (B) PEG precipitation, (C) $Al(OH)_3$ precipitation, (D) Ultra-centrifugation, (E) Ultra-filtration and (F) across all concentration methods.

Recovery Efficiency variation with concentration method

Table S5. Statistical values reported for all viruses comparing the recovery efficiency for the different concentration methods.

Concentration Methods Compared for All Viruses		***p* value**	**U**	**Cohen's d (95% CI)**
$Al(OH)_3$ Precipitation	Electronegative Membrane	**0.03 ***	4	1.658 (0.276 - 2.982)
	Flocculation	0.259	34	0.870 (-0.249 to 1.957)
	PEG Precipitation	0.209	35	0.845 (-0.271 to 1.929)
	Ultracentrifugation	0.479	47	0.425 (-0.541 to 1.377)
	Ultrafiltration	**0.021 ***	101	1.146 (0.202 to 2.068)
Electronegative Membrane Filtration	Flocculation	0.268	10.5	0.970 (-0.273 to 2.170)
	PEG Precipitation	0.639	14	0.367 (-0.802 to 1.526)
	Ultracentrifugation	**0.09**	12	1.030 (-0.109 to 2.136)
	Ultrafiltration	0.801	41	0.184 (-0.811 to 1.174)
Flocculation	PEG Precipitation	0.902	23	0.316 (-0.745 to 1.365)
	Ultracentrifugation	0.375	49	0.242 (-0.713 to 1.190)
	Ultrafiltration	0.141	38	0.586 (-0.309 to 1.469)
PEG Precipitation	Ultracentrifugation	0.596	45	0.484 (-0.485 to 1.439)
	Ultrafiltration	0.574	53.5	0.215 (-0.663 to 1.088)
Ultracentrifugation	Ultrafiltration	**0.061**	141	0.757 (-0.026 to 1.527)

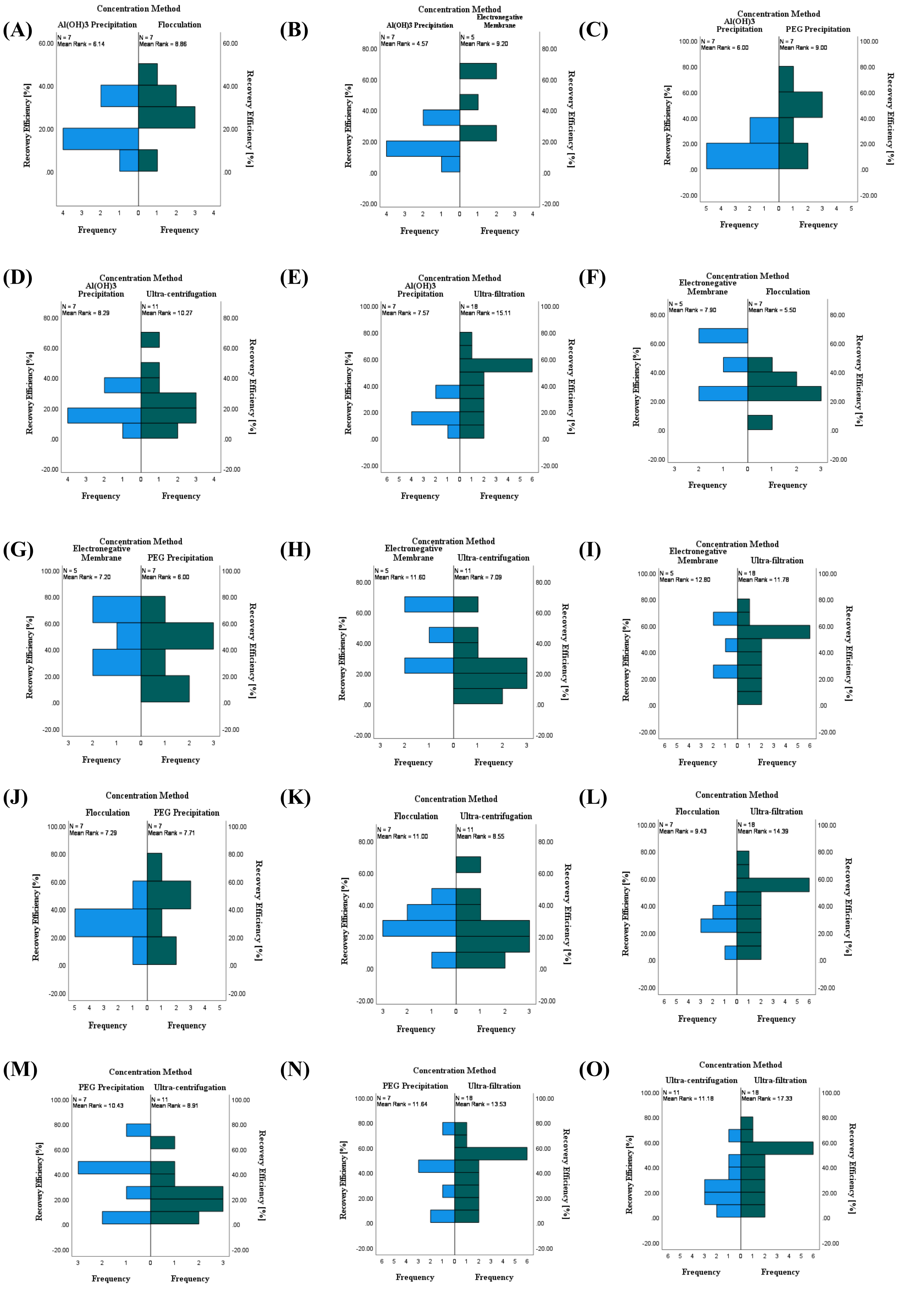

Figure S2. Histograms displaying the mean rank value for all viruses as paired comparison of the recovery efficiency for the different concentration methods.

Amplification Efficiency: Enveloped vs non-enveloped for each concentration method

Table S6. Statistical values for amplification efficiency of enveloped and non-enveloped virus groups for the different concentration methods.

Enveloped vs Non-enveloped Virus Compared for Concentration Methods	*p* value	U	Cohen's d (95% CI)
All methods	0.408	171.000	0.405 (-0.235 to 1.040)
$Al(OH)_3$ Precipitation	1.000	7.000	0.224 (-1.290 to 1.716)
Electronegative Membrane	**0.095**	10.000	1.282 (-0.581 to 3.044)
PEG Precipitation	0.905	9.000	0.319 (-1.017 to 1.633)
Ultrafiltration	**0.029 ***	14.000	1.072 (0.027 to 2.089)

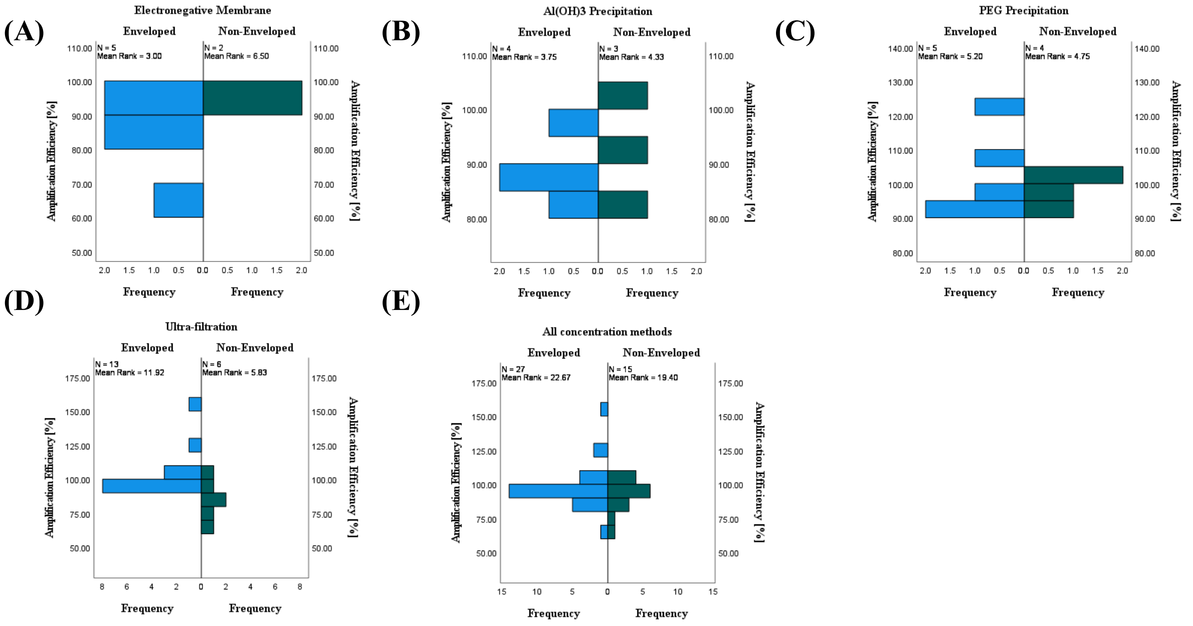

Figure S3. Histograms displaying the mean rank value for amplification efficiency of enveloped and non-enveloped virus groups compared for the different concentration methods: (A) Electronegative membrane filtration, (B) $Al(OH)_3$ precipitation, (C) PEG Precipitation, (D) Ultra-filtration, (E) Across all concentration methods.

Amplification Efficiency: Varied with concentration method

Table S7. Statistical values reported for all viruses comparing the amplification efficiency for various concentration methods specified.

Concentration Methods Compared for All Viruses		*p* value	U	Cohen's d (95% CI)
Al(OH)3 Precipitation	Electronegative Membrane	1.000	24.000	0.235 (-0.822 to 1.282)
	PEG Precipitation	**0.023** *	10.000	1.1108 (0.023 to 2.160)
	Ultrafiltration	0.135	40.500	0.477 (-0.404 to 1.349)
Electronegative Membrane	PEG Precipitation	**0.071**	14.000	1.164 (0.071 to 2.223)
	Ultrafiltration	0.209	44.000	0.593 (-0.296 to 1.469)
PEG Precipitation	Ultrafiltration	0.410	68.500	0.153 (-0.643 to 0.946)

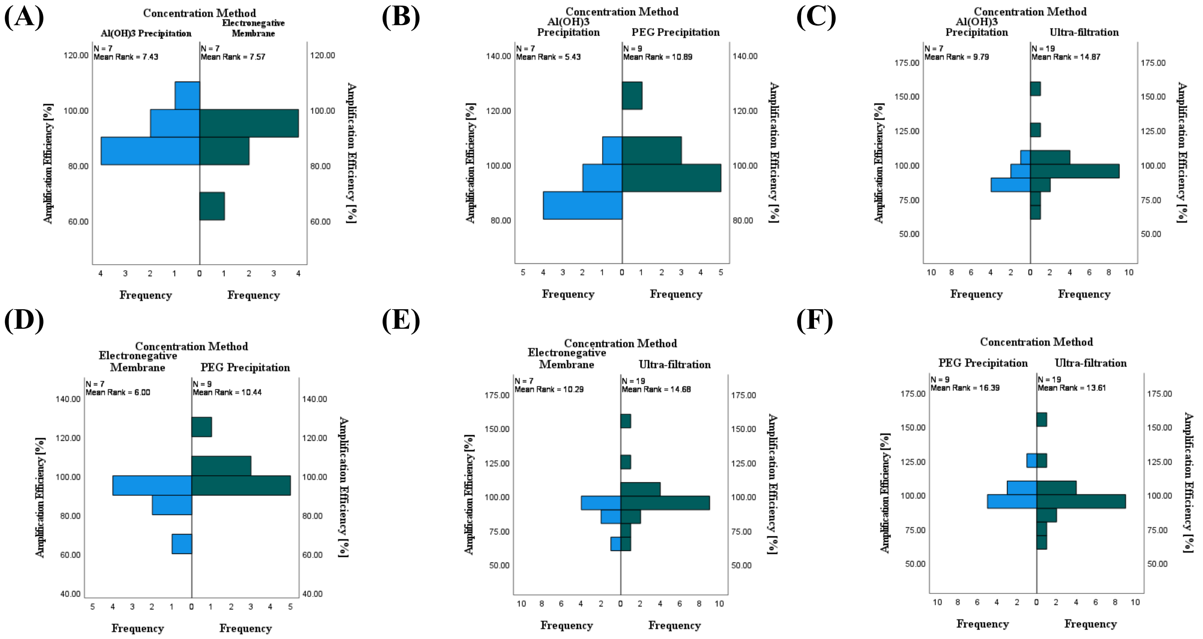

Figure S4. Histograms displaying the mean rank value for all viruses comparing the amplification efficiency for the different concentration methods: (A) Electronegative membrane filtration, (B) $Al(OH)_3$ precipitation, (C) PEG Precipitation, (D) Ultra-filtration, (E) Across all concentration methods.

Recovery Efficiency: Grab vs composite sampling

Table S8. Statistical values reported for recovery efficiency compared for composite and grab sampling.

Groups Compared for Sampling Type		*p* value	U	Cohen's d (95% CI)
Enveloped vs non-enveloped	Grab	1.000	120.500	0.014 (-0.691 to 0.718)
	Composite	0.333	11.000	1.157 (-0.558 to 2.802)
Enveloped	Grab vs Composite	0.441	21	0.705 (-0.803 to 2.190)
Non-enveloped	Grab vs Composite	0.308	40	0.380 (-0.520 to 1.271)
All viruses	Grab vs Composite	0.656	125	0.163 (-0.581 to 0.905)

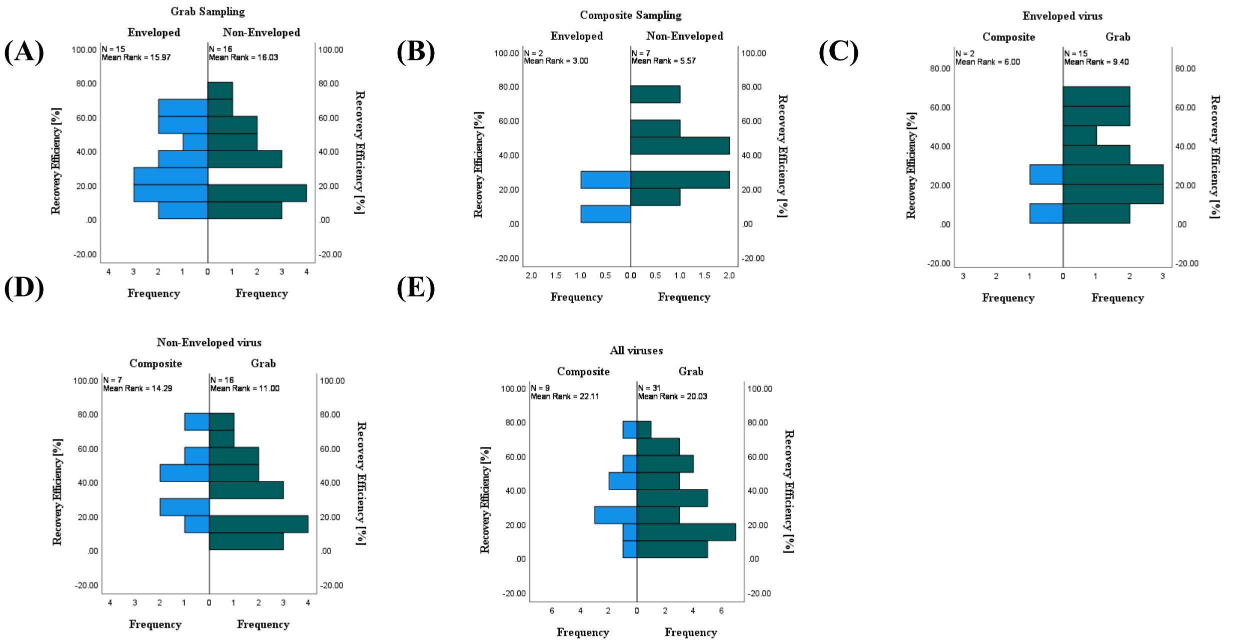

Figure S5. Histograms displaying the mean rank value for recovery efficiency compared for composite and grab sampling: (A) Grab sampling, (B) Composite sampling, (C) Enveloped viruses, (D) Non-enveloped viruses, (E) All viruses.

Recovery Efficiency: Use of chemical additives vs none

Table S9. Statistical values reported for recovery efficiency compared for usage of chemical additives during sample processing or without use of additives.

Groups Compared for Chemical Additive Usage		*p* value	U	Cohen's d (95% CI)
Enveloped vs non-enveloped groups compared	Addition of Chemicals	0.775	157.5	0.095 (-0.607 to 0.796)
	No Chemicals	0.387	68	0.503 (-0.374 to 1.368)
Enveloped	Addition of Chemicals vs None	0.705	61	0.222 (-0.640 to 1.078)
Non-enveloped	Addition of Chemicals vs None	0.116	198	0.606 (-0.113 to 1.317)
All viruses	Addition of Chemicals vs None	0.166	486.5	0.409 (-0.131 to 0.946)

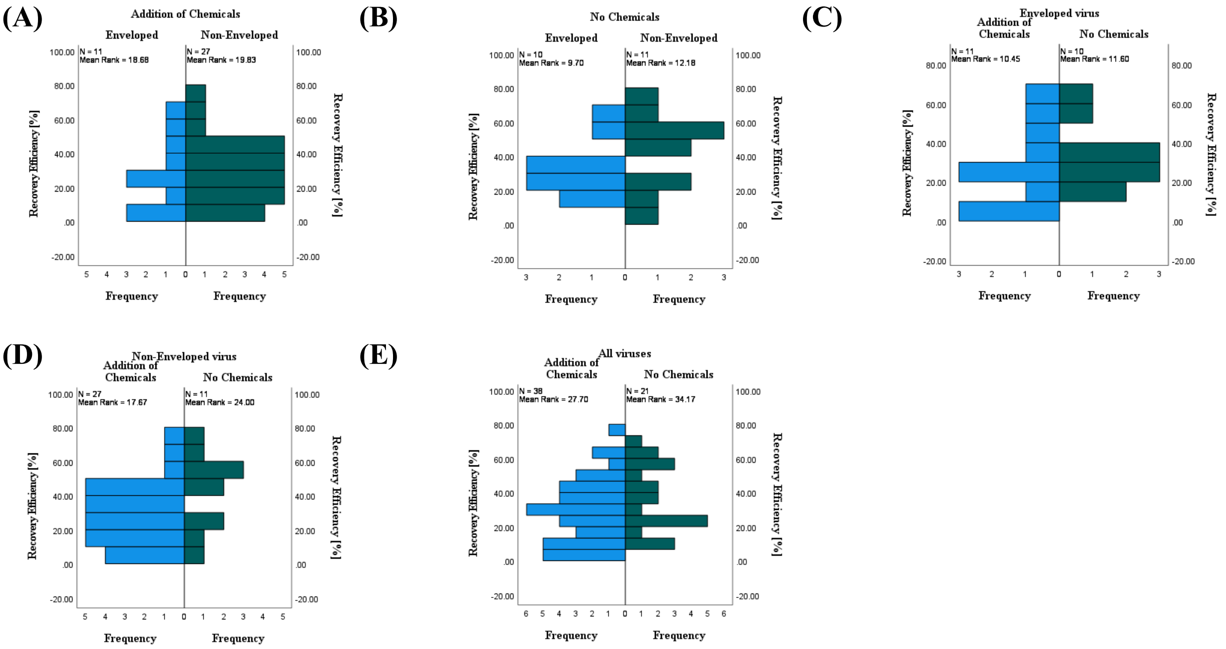

Figure S6. Histograms displaying the mean rank value for recovery efficiency compared for use of chemical additives vs no use of chemical additives: (A) Chemical additives, (B) No chemical additives, (C) Enveloped viruses, (D) Non-enveloped viruses, (E) All viruses.

Recovery Efficiency: Surrogates

Table S10. Statistical values reported for recovery efficiency compared for the specified virus groups.

Groups Compared for Specified Virus Types		*p* value	U	Cohen's d (95% CI)
Enveloped Surrogates	Non-enveloped Surrogates	0.468	263.5	0.202 (-0.417 to 0.819)
	Other Enveloped	0.294	23	0.826 (-0.691 to 2.317)
	Other Non-enveloped	0.681	67	0.311 (-0.555 to 1.171)
Non-enveloped Surrogates	Other Enveloped	0.417	43	0.672 (-0.773 to 2.106)
	Other Non-enveloped	0.695	136	0.148 (-0.631 to 0.925)
Other Enveloped	Other Non-enveloped	0.533	5	0.396 (-1.945 to 1.177)

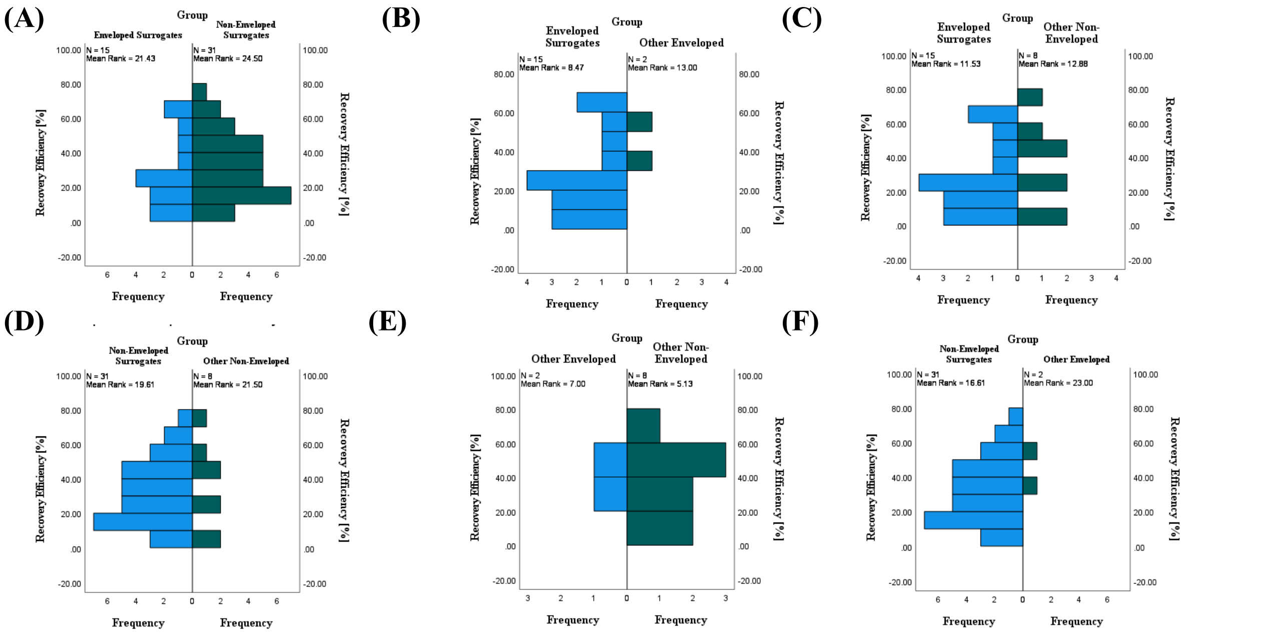

Figure S7. Histograms displaying the mean rank value for recovery efficiency compared for the specified virus groups.

References

[1] J. Zhang, K. Li, L. Zheng, J. Zhang, Z. Ren, T. Song, et al., *Front Cell Infect Microbiol.* **2020**, *10*, 558472. DOI: 10.3389/fcimb.2020.558472

[2] Y. Zhang, C. Chen, Y. Song, S. Zhu, D. Wang, H. Zhang, et al., *Emerg Microbes Infect.* **2020**, *9(1)*, 2501-8. DOI: 10.1080/22221751.2020.1844551

[3] O. Turriziani, I. Sciandra, L. Mazzuti, D. Di Carlo, C. Bitossi, M. Calabretto, et al., *J Med Virol.* **2021**, *93(2)*, 886-91. DOI: 10.1002/jmv.26332

[4] M. Masiá, G. Telenti, M. Fernández, J. A. García, V. Agulló, S. Padilla, et al., *Open Forum Infect Dis.* **2021**, *8(2)*. DOI: 10.1093/ofid/ofab005

[5] G. Q. Chen, W. T. Luo, C. H. Zhao, C. N. Li, Z. S. Hong, J. Y. Xia, et al., *J Infect Dev Ctries* **2020**, *14(8)*, 847-52. DOI: 10.3855/jidc.12885

[6] S. Zheng, J. Fan, F. Yu, B. Feng, B. Lou, Q. Zou, et al., *BMJ.* **2020**, *369*, m1443. DOI: 10.1136/bmj.m1443

[7] X. S. Wei, X. Wang, Y. R. Niu, L. L. Ye, W. B. Peng, Z. H. Wang, et al., *Clin Gastroenterol Hepatol.* **2020**, *18(8)*, 1753-9.e2. DOI: 10.1016/j.cgh.2020.04.030

[8] Y. Wu, C. Guo, L. Tang, Z. Hong, J. Zhou, X. Dong, et al., *Lancet Gastroenterol Hepatol.* **2020**, *5(5)*, 434-5. DOI: 10.1016/S2468-1253(20)30083-2

[9] Y. Lu, Y. Li, Y. Wang, J. Luo, W. Yu, *Int Immunopharmacol.* **2020**, *89*. DOI: 10.1016/j.intimp.2020.107089

[10] F. Xiao, M. Tang, X. Zheng, Y. Liu, X. Li, H. Shan, *Gastroenterology.* **2020**, *158(6)*, 1831-3.e3. DOI: 10.1053/j.gastro.2020.02.055

[11] L. Lin, X. Jiang, Z. Zhang, S. Huang, Z. Zhang, Z. Fang, et al., *Gut.* **2020**, *69(6)*, 997-1001. DOI: 10.1136/gutjnl-2020-321013

[12] C. Chen, G. Gao, Y. Xu, L. Pu, Q. Wang, L. Wang, et al., *Ann Intern Med.* **2020**, *172(12)*, 832-4. DOI: 10.7326/m20-0991

[13] W. Deng, T. W. Guang, M. Yang, J. R. Li, D. P. Jiang, C. Y. Li, et al., *BMC Infect Dis.* **2020**, *20(1)*. DOI: 10.1186/s12879-020-05151-y

[14] K. S. Cheung, I. F. N. Hung, P. P. Y. Chan, K. C. Lung, E. Tso, R. Liu, et al., *Gastroenterology.* **2020**, *159(1)*, 81-95. DOI: 10.1053/j.gastro.2020.03.065

[15] X. Lu, L. Wang, S. K. Sakthivel, B. Whitaker, J. Murray, S. Kamili, et al., *Emerg Infect Dis.* **2020**, *26(8)*, 1654-65. DOI: 10.3201/eid2608.201246

[16] W. A. Szymczak, D. Y. Goldstein, E. P. Orner, R. A. Fecher, R. T. Yokoda, K. A. Skalina, et al., *J Clin Microbiol.* **2020**, *58(9)*. DOI: 10.1128/jcm.01369-20

[17] Y. Chen, L. Chen, Q. Deng, G. Zhang, K. Wu, L. Ni, et al., *J Med Virol.* **2020**, *92(7)*, 833-40. DOI: 10.1002/jmv.25825

[18] C. Z. Hua, Z. P. Miao, J. S. Zheng, Q. Huang, Q. F. Sun, H. P. Lu, et al., *J Med Virol.* **2020**, *92(11)*, 2804-12. DOI: 10.1002/jmv.26180

[19] W. Chen, Y. Lan, X. Yuan, X. Deng, Y. Li, X. Cai, et al., *Emerg Microbes Infect.* **2020**, *9(1)*, 469-73. DOI: 10.1080/22221751.2020.1732837

[20] F. Xiao, J. Sun, Y. Xu, F. Li, X. Huang, H. Li, et al., *Emerg Infect Dis.* **2020**, *26(8)*, 1920-2. DOI: 10.3201/eid2608.200681

[21] J. Stohr, M. Wennekes, M. van der Ent, B. M. W. Diederen, M. F. Q. Kluytmans-van den Bergh, A. M. C. Bergmans, et al., *J Clin Virol.* **2020**, *133*, 104686. DOI: 10.1016/j.jcv.2020.104686

[22] Z. Xue, H. You, Y. Luan, H. Chen, Q. Du, Y. Han, et al., *Chin J Microbiol Immunol.* **2020**, *40(8)*, 569-73. DOI: 10.3760/cma.j.cn112309-20200524-00274

[23] C. Han, C. Duan, S. Zhang, B. Spiegel, H. Shi, W. Wang, et al., *Am J Gastroenterol.* **2020**, *115(6)*, 916-23. DOI: 10.14309/ajg.0000000000000664

[24] Y. He, J. Luo, J. Yang, J. Song, L. Wei, W. Ma, *Front Cell Infect Microbiol.* **2020**, *10*. DOI: 10.3389/fcimb.2020.00445

[25] Q. J. Wang, Y. Z. Yao, J. S. Song, Q. Wang, L. Y. Xu, Z. J. Bao, et al., *BMC Infect Dis.* **2020**, *20(1)*. DOI: 10.1186/s12879-020-05549-8

[26] Y. Pan, D. Zhang, P. Yang, L. L. M. Poon, Q. Wang, *Lancet Infect Dis.* **2020**, *20(4)*, 411-2. DOI: 10.1016/S1473-3099(20)30113-4

[27] A. Mesoraca, K. Margiotti, A. Viola, A. Cima, D. Sparacino, C. Giorlandino, *Virol J.* **2020**, *17(1)*, 86. DOI: 10.1186/s12985-020-01359-1

[28] W. Zhang, R.-H. Du, B. Li, X.-S. Zheng, X.-L. Yang, B. Hu, et al., *Emerg Microbes Infect*. **2020**, *9(1)*, 386-9. DOI: 10.1080/22221751.2020.1729071

[29] T. Zuo, F. Zhang, G. C. Y. Lui, Y. K. Yeoh, A. Y. L. Li, H. Zhan, et al., *Gastroenterology*. **2020**, *159(3)*, 944-55.e8. DOI: 10.1053/j.gastro.2020.05.048

[30] J. Zhang, S. Wang, Y. Xue, *J Med Virol*. **2020**, *92(6)*, 680-2. DOI: 10.1002/jmv.25742

[31] Y. Li, Y. Hu, Y. Yu, X. Zhang, B. Li, J. Wu, et al., *J Med Virol*. **2020**, *92(10)*, 1938-47. DOI: 10.1002/jmv.25905

[32] N. Zhang, Y. Gong, F. Meng, Y. Shi, J. Wang, P. Mao, et al., *Sci China Life Sci*. **2020**, 1-3. DOI: 10.1007/s11427-020-1783-9

[33] S. Li, W. Jiang, J. Huang, Y. Liu, L. Ren, L. Zhuang, et al., *Eur Respir J*. **2020**, *56(6)*. DOI: 10.1183/13993003.02060-2020

[34] Y. Xu, X. Li, B. Zhu, H. Liang, C. Fang, Y. Gong, et al., *Nat Med*. **2020**, *26(4)*, 502-5. DOI: 10.1038/s41591-020-0817-4

[35] L. Peng, J. Liu, W. Xu, Q. Luo, D. Chen, Z. Lei, et al., *J Med Virol*. **2020**, *92(9)*, 1676-80. DOI: 10.1002/jmv.25936

[36] R. Wölfel, V. M. Corman, W. Guggemos, M. Seilmaier, S. Zange, M. A. Müller, et al., *Nature*. **2020**, *581(7809)*, 465-9. DOI: 10.1038/s41586-020-2196-x

[37] C. Xie, L. Jiang, G. Huang, H. Pu, B. Gong, H. Lin, et al., *Int J Infect Dis*. **2020**, *93*, 264-7. DOI: 10.1016/j.ijid.2020.02.050

[38] B. E. Young, S. W. X. Ong, S. Kalimuddin, J. G. Low, S. Y. Tan, J. Loh, et al., *JAMA*. **2020**, *323(15)*, 1488-94. DOI: 10.1001/jama.2020.3204

[39] C. Jiehao, X. Jin, L. Daojiong, Y. Zhi, X. Lei, Q. Zhenghai, et al., *Clin Infect Dis*. **2020**, *71(6)*, 1547-51. DOI: 10.1093/cid/ciaa198

[40] F. X. Lescure, L. Bouadma, D. Nguyen, M. Parisey, P. H. Wicky, S. Behillil, et al., *Lancet Infect Dis*. **2020**, *20(6)*, 697-706. DOI: 10.1016/S1473-3099(20)30200-0

[41] G. T. Lin, Y. H. Zhang, M. F. Xiao, Y. Wei, J. N. Chen, D. J. Lin, et al., *Health Inf Sci Syst*. **2021**, *9(1)*. DOI: 10.1007/s13755-020-00136-2

[42] T. Prado, T. M. Fumian, C. F. Mannarino, P. C. Resende, F. C. Motta, A. L. F. Eppinghaus, et al., *Water Res*. **2021**, *191*, 116810. DOI: 10.1016/j.watres.2021.116810

[43] H. Mlejnkova, K. Sovova, P. Vasickova, V. Ocenaskova, L. Jasikova, E. Juranova, *Int J Environ Res Public Health*. **2020**, *17(15)*, 1-9. DOI: 10.3390/ijerph17155508

[44] A. Hata, H. Hara-Yamamura, Y. Meuchi, S. Imai, R. Honda, *Sci Total Environ*. **2021**, *758*. DOI: 10.1016/j.scitotenv.2020.143578

[45] W. Randazzo, P. Truchado, E. Cuevas-Ferrando, P. Simon, A. Allende, G. Sanchez, *Water Res*. **2020**, *181*. DOI: 10.1016/j.watres.2020.115942

[46] G. La Rosa, P. Mancini, G. Bonanno Ferraro, C. Veneri, M. Iaconelli, L. Bonadonna, et al., *Sci Total Environ*. **2021**, *750*. DOI: 10.1016/j.scitotenv.2020.141711

[47] I. Bar-Or, K. Yaniv, M. Shagan, E. Ozer, M. Weil, V. Indenbaum, et al., *Frontiers in Public Health*. **2022**, *9*. DOI: 10.3389/fpubh.2021.561710

[48] S. Arora, A. Nag, J. Sethi, J. Rajvanshi, S. Saxena, S. K. Shrivastava, et al., *Water Sci Technol*. **2020**, *82(12)*, 2823-36. DOI: 10.2166/wst.2020.540

[49] G. Medema, L. Heijnen, G. Elsinga, R. Italiaander, A. Brouwer, *Environ Sci Technol Lett*. **2020**, *7(7)*, 511-6. DOI: 10.1021/acs.estlett.0c00357

[50] W. Randazzo, E. Cuevas-Ferrando, R. Sanjuan, P. Domingo-Calap, G. Sanchez, *Int J Hyg Environ Health*. **2020**, *230*. DOI: 10.1016/j.ijheh.2020.113621

[51] G. La Rosa, M. Iaconelli, P. Mancini, G. Bonanno Ferraro, C. Veneri, L. Bonadonna, et al., *Sci Total Environ*. **2020**, *736*. DOI: 10.1016/j.scitotenv.2020.139652

[52] W. Ahmed, N. Angel, J. Edson, K. Bibby, A. Bivins, J. W. O'Brien, et al., *Sci Total Environ*. **2020**, *728*. DOI: 10.1016/j.scitotenv.2020.138764

[53] S. P. Sherchan, S. Shahin, L. M. Ward, S. Tandukar, T. G. Aw, B. Schmitz, et al., *Sci Total Environ*. **2020**, *743*. DOI: 10.1016/j.scitotenv.2020.140621

[54] J. Martin, D. Klapsa, T. Wilton, M. Zambon, E. Bentley, E. Bujaki, et al., *Viruses*. **2020**, *12(10)*. DOI: 10.3390/v12101144

[55] M. H. Jafferali, K. Khatami, M. Atasoy, M. Birgersson, C. Williams, Z. Cetecioglu, *Sci Total Environ*. **2021**, *755*. DOI: 10.1016/j.scitotenv.2020.142939

[56] S. G. Rimoldi, F. Stefani, A. Gigantiello, S. Polesello, F. Comandatore, D. Mileto, et al., *Sci Total Environ*. **2020**, *744*. DOI: 10.1016/j.scitotenv.2020.140911

[57] Y. Ye, R. M. Ellenberg, K. E. Graham, K. R. Wigginton, *Environ Sci Technol*. **2016**, *50(10)*, 5077-85. DOI: 10.1021/acs.est.6b00876
[58] K. E. Graham, S. K. Loeb, M. K. Wolfe, D. Catoe, N. Sinnott-Armstrong, S. Kim, et al., *Environmental Science and Technology*. **2021**, *55(1)*, 488-98. DOI: 10.1021/acs.est.0c06191
[59] L. Heijnen, G. Medema, *J Water Health*. **2011**, *9(3)*, 434-42. DOI: 10.2166/wh.2011.019
[60] W. Ahmed, P. M. Bertsch, A. Bivins, K. Bibby, K. Farkas, A. Gathercole, et al., *Sci Total Environ*. **2020**, *739*, 139960. DOI: 10.1016/j.scitotenv.2020.139960
[61] M. Hemalatha, U. Kiran, S. K. Kuncha, H. Kopperi, C. G. Gokulan, S. V. Mohan, et al., *Sci Total Environ*. **2021**, *768*. DOI: 10.1016/j.scitotenv.2020.144704
[62] S. Westhaus, F. A. Weber, S. Schiwy, V. Linnemann, M. Brinkmann, M. Widera, et al., *Sci Total Environ*. **2021**, *751*. DOI: 10.1016/j.scitotenv.2020.141750
[63] A. Nemudryi, A. Nemudraia, T. Wiegand, K. Surya, M. Buyukyoruk, C. Cicha, et al., *Cell Rep*. **2020**, *1(6)*, 100098. DOI: 10.1016/j.xcrm.2020.100098
[64] A. M. Hokajärvi, A. Rytkönen, A. Tiwari, A. Kauppinen, S. Oikarinen, K. M. Lehto, et al., *Sci Total Environ*. **2021**, *770*. DOI: 10.1016/j.scitotenv.2021.145274
[65] F. Wu, J. Zhang, A. Xiao, X. Gu, W. L. Lee, F. Armas, et al., *mSystems*. **2020**, *5(4)*. DOI: 10.1128/mSystems.00614-20
[66] S. Agrawal, L. Orschler, S. Lackner, *Sci Rep*. **2021**, *11(1)*, 5372. DOI: 10.1038/s41598-021-84914-2
[67] X. W. Wang, J. S. Li, T. K. Guo, B. Zhen, Q. X. Kong, B. Yi, et al., *J Virol Methods*. **2005**, *128(1-2)*, 156-61. DOI: 10.1016/j.jviromet.2005.03.022
[68] H. Mlejnkova, K. Sovova, P. Vasickova, V. Ocenaskova, L. Jasikova, E. Juranova, *Int J Environ Res Public Health*. **2020**, *17(15)*. DOI: 10.3390/ijerph17155508
[69] M. Rusiñol, S. Martínez-Puchol, E. Forés, M. Itarte, R. Girones, S. Bofill-Mas, *Curr Opin Environ Sci Health*. **2020**, *17*, 21-8. DOI: 10.1016/j.coesh.2020.08.002
[70] Y. Qiu, B. E. Lee, N. J. Ruecker, N. Neumann, N. Ashbolt, X. Pang, *J Virol Methods*. **2016**, *237*, 150-3. DOI: 10.1016/j.jviromet.2016.09.010
[71] H. Amdiouni, L. Maunula, K. Hajjami, A. Faouzi, A. Soukri, J. Nourlil, *Curr Microbiol*. **2012**, *65(4)*, 432-7. DOI: 10.1007/s00284-012-0174-8
[72] K. Farkas, D. M. Cooper, J. E. McDonald, S. K. Malham, A. de Rougemont, D. L. Jones, *Sci Total Environ*. **2018**, *634*, 1174-83. DOI: 10.1016/j.scitotenv.2018.04.038
[73] T. Miura, S. Lhomme, J.-C. Le Saux, P. Le Mehaute, Y. Guillois, E. Couturier, et al., *Food Environ Virol*. **2016**, *8(3)*, 194-9. DOI: 10.1007/s12560-016-9241-9
[74] A. K. da Silva, F. S. Le Guyader, J. C. Le Saux, M. Pommepuy, M. A. Montgomery, M. Elimelech, *Environ Sci Technol*. **2008**, *42(24)*, 9151-7. DOI: 10.1021/es802787v
[75] W. Randazzo, J. Piqueras, Z. Evtoski, G. Sastre, R. Sancho, C. Gonzalez, et al., *Food Environ Virol*. **2019**, *11(4)*, 350-63. DOI: 10.1007/s12560-019-09392-2
[76] W. Randazzo, M. Khezri, J. Ollivier, F. S. Le Guyader, J. Rodríguez-Díaz, R. Aznar, et al., *Int J Food Microbiol*. **2018**, *266*, 1-7. DOI: 10.1016/j.ijfoodmicro.2017.11.011
[77] B. W. Schmitz, M. Kitajima, M. E. Campillo, C. P. Gerba, I. L. Pepper, *Environ Sci Technol*. **2016**, *50(17)*, 9524-32. DOI: 10.1021/acs.est.6b01384
[78] M. Muscillo, M. Fratini, R. Graffeo, M. Sanguinetti, V. Martella, K. Y. Green, et al., *Food Environ Virol*. **2013**, *5(4)*, 194-202. DOI: 10.1007/s12560-013-9121-5
[79] A. Hata, M. Kitajima, H. Katayama, *J Appl Microbiol*. **2013**, *114(2)*, 545-54. DOI: 10.1111/jam.12051
[80] M. Amarasiri, M. Kitajima, A. Miyamura, R. Santos, S. Monteiro, T. Miura, et al., *Int J Hyg Environ Health*. **2018**, *221(3)*, 578-85. DOI: 10.1016/j.ijheh.2018.02.008
[81] T. M. Fumian, J. M. Fioretti, J. H. Lun, I. A. L. dos Santos, P. A. White, M. P. Miagostovich, *Environ Int*. **2019**, *123*, 282-91. DOI: 10.1016/j.envint.2018.11.054
[82] M. A. Laverick, A. P. Wyn-Jones, M. J. Carter, *Lett Appl Microbiol*. **2004**, *39(2)*, 127-36. DOI: 10.1111/j.1472-765X.2004.01534.x
[83] B. Calgua, T. Fumian, M. Rusiñol, J. Rodriguez-Manzano, V. A. Mbayed, S. Bofill-Mas, et al., *Water Res*. **2013**, *47(8)*, 2797-810. DOI: 10.1016/j.watres.2013.02.043
[84] E. M. Symonds, C. Sinigalliano, M. Gidley, W. Ahmed, S. M. McQuaig-Ulrich, M. Breitbart, *J Appl Microbiol*. **2016**, *121(5)*, 1469-81. DOI: 10.1111/jam.13252
[85] H. Katayama, E. Haramoto, K. Oguma, H. Yamashita, A. Tajima, H. Nakajima, et al., *Water Res*. **2008**, *42(6-7)*, 1441-8. DOI: 10.1016/j.watres.2007.10.029

[86] T. M. Fumian, J. P. Leite, A. A. Castello, A. Gaggero, M. S. Caillou, M. P. Miagostovich, *J Virol Methods*. **2010**, *170(1-2)*, 42-6. DOI: 10.1016/j.jviromet.2010.08.017
